# 7-Aminocoumarin-4-acetic Acid as a Fluorescent Probe for Detecting Bacterial Dipeptidyl Peptidase Activities in Water-in-Oil Droplets and in Bulk

**DOI:** 10.1021/acs.analchem.1c04108

**Published:** 2021-12-28

**Authors:** Akihiro Nakamura, Nobuyuki Honma, Yuma Tanaka, Yoshiyuki Suzuki, Yosuke Shida, Yuko Tsuda, Koushi Hidaka, Wataru Ogasawara

**Affiliations:** † Department of Science of Technology Innovation, Nagaoka University of Technology, 1603-1 Kamitomioka, Nagaoka, Niigata 940-2188, Japan; ‡ Department of Bioengineering, Nagaoka University of Technology, 1603-1 Kamitomioka, Nagaoka, Niigata 940-2188, Japan; § Faculty of Pharmaceutical Sciences, Cooperative Research Center of Life Sciences, Kobe Gakuin University, 1-1-3 Minatojima, Chuo-ku, Kobe, Hyogo 650-8586, Japan; ∥ Graduate School of Health Sciences, Kobe University, 7-10-2 Tomogaoka, Suma-ku, Kobe, Hyogo 654-0142, Japan

## Abstract

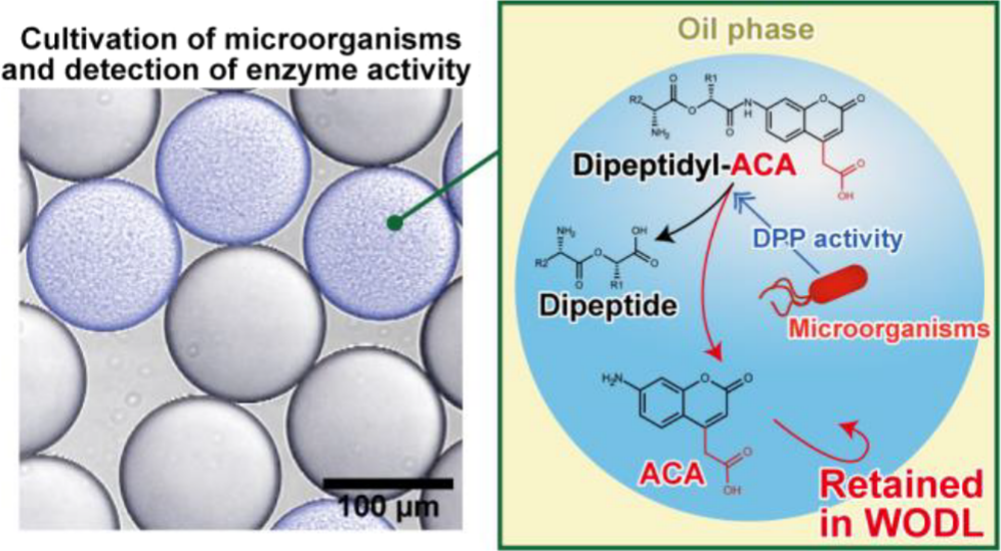

Droplet-based microfluidic systems are a powerful tool for biological assays with high throughput. Water-in-oil droplets (WODLs) are typically used in droplet-based microfluidic systems to culture microorganisms and perform enzyme assays. However, because of the oil surrounding the nanoliter and picoliter volumes of WODLs, availability of suitable substrates is limited. For instance, although 7-amino-4-methylcoumarin (AMC) is commonly used as a fluorescent probe of the substrate to detect peptidase activity, AMC leaks from WODLs to the oil phase due to its high hydrophobicity. Thus, AMC substrates cannot be used in droplet-based microfluidic systems with WODLs. In this study, we developed a peptidase substrate consisting of a dipeptide and 7-aminocoumarin-4-acetic acid (ACA), an AMC-derived fluorogenic compound. ACA was retained in the WODL for more than 7 days, and the dipeptidyl ACA substrate detected dipeptidyl peptidase (DPP) activity in the WODL. Compared to AMC substrates, the substrate specificity constants of DPPs for ACA substrates increased up to 4.7-fold. Fluorescence-activated droplet sorting made high-throughput screening of microorganisms based on DPP activity using the dipeptidyl ACA substrate possible. Since ACA could be applied to various substrates as a fluorescent probe, detectable microbial enzyme activities for droplet-based microfluidic systems can be largely expanded.

Droplet-based microfluidic systems are a powerful tool for biological assays with high throughput.^1,2^ Along with high throughput, compartmentalization and miniaturization of microbial cultivation are features specific to this technique,^3^ which facilitate the screening of microorganisms based on enzyme activity.^4^ After screening one million species, it has been reported that the number of microorganisms possessing the targeting activity for activity-based screening of microorganisms is 10 species or fewer.^5^ Thus, high-throughput screening offers significant advantages over the low acquisition rate of viable microorganisms. In addition, stochastic compartmentalization of microorganisms is effective in isolating a single cell from a mixed suspension of various microorganisms. Miniaturization also reduces the required amounts of media and substrates, which reduces costs. Thus, droplet-based microfluidic systems have recently been studied for their application in screening of microorganisms.

In droplet-based microfluidic systems, the culturing of microorganisms and enzyme assays are typically performed inside nanoliter-sized or picoliter-sized water-in-oil droplets (WODLs).^6−8^ To date, various microorganisms have been screened based on enzyme activities of amylase, glucosidase, esterase, lipase, cellulase, and laccase.^7,9−14^ However, the scope of application of WODLs to droplet-based microfluidic systems is limited to some bacterial enzyme activities. This is because the most significant feature of the WODL, which is not found in other enzyme assay methods, is the existence of the oil phase surrounding the nanoliter or picoliter volumes of aqueous solution. This feature is sometimes disadvantageous in detecting enzyme activity in a WODL, especially when used with fluorogenic substrates, which are generally used to detect enzyme activity in a WODL with fluorescence-activated droplet sorting (FADS).^12,15^ Because the hydrophobic fluorogenic substrate leaks from the aqueous solution to the oil phase and diffuses throughout WODLs, the evaluation of enzyme activity is difficult.^16−18^ Hence, the fluorogenic substrate used to detect enzyme activity in a WODL needs hydrophilicity. To prevent compound leakage from a WODL, methods, such as addition of hydrophilic functional groups (phosphate, carboxyl, and sulfo groups) to compounds^16,17,19^ and alteration of types and concentrations of surfactants,^18,20^ have been investigated.

Coumarins are commonly used as fluorescent probes of the substrate to detect enzyme activity, in particular protease activity. Unsubstituted coumarins show almost no fluorescence, but when an electron-donating group is introduced at position 7, they exhibit strong luminescence.^21^ In fact, by introducing a hydroxy group or amino group at position 7, they can be bound to various substrates, such as amino acids, lipids, sugars, and other molecules.^22,23^ 7-Amino-4-methylcoumarin (AMC), with the addition of the amino group at position 7, has blue fluorescence and has been widely used as a probe for the fluorogenic substrate.^23^ However, a previous study by Woronoff et al. showed that AMC leaked into the oil phase and could not be used in a WODL.^17^ They added a sulfo group to AMC to prevent compound leakage from the WODL and succeeded in detecting the acylase activity of *E. coli* in the WODL.^17^ Therefore, coumarin derivatives retained in the WODL can facilitate high-throughput screening of microorganisms based on their enzyme activity using droplet-based microfluidic systems.

In this study, we selected dipeptidyl peptidases (DPP) for targeted bacterial enzyme activity. DPP possesses hydrolase activity, releasing the dipeptide from the N-terminus of oligopeptide. In particular, dipeptidyl peptidases 7 (DPP7s) and 11 (DPP11s) belong to the family S46 peptidases and are distributed in some bacteria; approximately 540 species (MEROPS database), mainly under Proteobacteria and Bacteroides, were used for demonstration purposes.^24,25^ DPP7s exhibit a broad substrate specificity for aliphatic and aromatic residues at the P1 position (NH_2_–P2–P1–P1′–P2′–..., where the P1–P1′ bond is the scissile bond), whereas DPP11s exhibit a strict substrate specificity for acidic residues (Asp/Glu) at the P1 position.

Here, we developed an AMC-derived substrate retained in a WODL that can be used in droplet-based microfluidic systems and demonstrated the availability of an AMC-derived substrate to screen microorganisms based on DPP activities. We synthesized a dipeptidyl 7-aminocoumarin-4-acetic acid (ACA) substrate in which the carboxyl group of the dipeptide forms a peptide bond with the amino group at position 7 of ACA.^26^ ACA was retained in the WODL for more than 7 days, and the dipeptidyl ACA substrate detected the DPP activity of the bacterial cell in the WODL in addition to detecting purified DPP activity. Since ACA can be applied not only to DPP but to various other substrates, detectable microbial enzyme activity for droplet-based microfluidic systems can be greatly expanded.

## Experimental Section

### Materials, Bacterial Strains, Medium, and Enzymes

A fluorescent nucleic acid probe for bacterial sorting (FNAP-sort),^15^ which is an RNA probe labeled with Alexa 488 at the 5′ end and with Black Hole Quencher1 (BHQ1) at the 3′ end, was synthesized by Eurofins Genomics (Japan). 7-Amino-4-methyl-3-coumarinylacetic acid (AMCA-H), 7-amino-4-trifluoromethylcoumarin (AFC), and sulforhodamine B were purchased from FUJIFILM Wako Pure Chemical Corporation (Japan). AMC and ACA were purchased from Peptide Institute, Inc. (Japan) and Fluorochem Ltd. (UK), respectively. 7-Aminocoumarin-4-methanesulfonic acid (ACMS) was synthesized as described in the Supplementary Information. l-Methionyl-l-leucyl-7-aminocoumarin-4-acetic acid (Met–Leu–ACA), l-methionyl-l-leucyl-7-aminocoumarin-4-methanesulfonic acid (Met–Leu–ACMS), and l-leucyl-l-aspartyl-7-aminocoumarin-4-acetic acid (Leu–Asp–ACA) were synthesized in this study (see details in the Supporting Information). l-Methionyl-l-leucyl-7-amino-4-methylcoumarin (Met–Leu–AMC) and l-leucyl-l-aspartyl-7-amino-4-methylcoumarin (Leu–Asp–AMC) were purchased from Peptide Institute, Inc. (Japan). Two types of bacteria were used in this study: *Escherichia coli* DH5α (Takara Bio Inc., Japan) derived from the K12 strain was transfected with a plasmid (p005-RFP-strong, Addgene, MA, USA) containing a red fluorescent protein (RFP)-coding gene and used as a DPP-nonproducing bacterium. *Pseudoxanthomonas mexicana* WO24, isolated by Ogasawara, W,^27^ was used as a DPP-producing bacterium. *Tofu* (soybean curd) factory waste fluid was sampled from *Imai-tofu-ten* at Nagaoka city, Niigata, Japan. The casitone medium comprised 1% (w/v) Bacto Casitone, 0.2% (w/v) Bacto Yeast extract, and 4 mM MgSO_4_. The LB agar medium comprised 1% (w/v) Bacto Tryptone, 0.5% (w/v) Bacto Yeast extract, 1% (w/v) NaCl, and 1.5% (w/v) agar. Dipeptidyl aminopeptidase BII from *P. mexicana* WO24 (PmDAP BII), DPP7 from *Stenotrophomonas maltophilia* (SmDPP7), DPP11 from *S. maltophilia* (SmDPP11), and DPP11 from *Porphyromonas gingivalis* (PgDPP11) were overexpressed and purified as described in the literature.^25,28,29^

### WODL Generation and Sorting

WODLs with a diameter of about 120 μm were produced using an On-Chip droplet generator (On-Chip Biotechnologies, Japan) at a rate of about 400,000 droplets/min. The oil phase was HFE-7500 3 M Novec Engineered Fluid (HFE-7500) containing 2% (w/w) 008-FluoroSurfactant (RAN Biotechnologies, MA, USA) as a surfactant. FADS was performed using an On-Chip Sort (On-Chip Biotechnologies, Japan) at a maximum rate of about 300 droplets/s. A quantity of 0.1% (w/w) 008-FluoroSurfactant in HFE-7500 was used as sheath solution.

### Retention Time Characterization in the WODL

Each fluorescent substance was diluted to 100 μM with 50 mM sodium phosphate buffer containing 5 mM EDTA after being dissolved in dimethyl sulfoxide at 10 mM. The excitation and emission light spectra of each coumarin derivative are shown in Figure S1. A WODL with fluorescent substances (positive WODL) and a WODL without fluorescent substances (negative WODL) were prepared, and equal volumes of both WODLs were mixed. The negative WODLs contained sulforhodamine B. Blue and red fluorescence intensities were measured with a confocal microscope and analyzed by ImageJ, as described below. MiLog*P* values were calculated by Molinspiration (http://www.molinspiration.com//cgi-bin/properties).

### Image Analysis

Micrographs were obtained with a laser-scanning confocal microscope system A1 (Nikon, Japan) operated by NIS-Elements software (Nikon, Japan), under 100× magnification. The WODLs were placed into a μ-Slide VI flat microscopy chamber (Ibidi, Germany) prefilled with 0.1% (w/w) 008-FluoroSurfactant in HFE-7500. A fluorescence intensity analysis was performed with ImageJ.^30^ The calibration curve of the fluorescence intensity is shown in Figure S2.

### Determination of Kinetics Parameters toward the Dipeptidyl Substrate

Kinetic parameters were determined by fitting the experimental data to the Michaelis–Menten equation using Excel Solver (Microsoft, WA, USA) by nonlinear least-squares fitting with various substrate concentrations: Met–Leu–AMC (0.781, 1.56, 3.13, 6.25, 12.5, 25, 50, and 100 μM for 2.5 nM PmDAPBII and 2.5 nM SmDPP7); Met–Leu–ACA (0.195, 0.391, 0.781, 1.56, 3.13, 6.25, 12.5, and 25 μM for 2 nM PmDAPBII and 0.391, 0.781, 1.56, 3.13, 6.25, 12.5, 25, and 50 μM for 4 nM SmDPP7); Met–Leu–ACMS (0.781, 1.56, 3.13, 6.25, 12.5, 25, 50, and 100 μM for 500 nM PmDAPBII, and 100 nM SmDPP7); Leu–Asp–AMC (0.781, 1.56, 3.13, 6.25, 12.5, 25, 50, and 100 μM for 0.5 nM PgDPP11, and 0.2 nM SmDPP11); and Leu–Asp–ACA (0.781, 1.56, 3.13, 6.25, 12.5, 25, 50, and 100 μM for 0.5 nM PgDPP11, and 0.1 nM SmDPP11). The enzyme reaction was performed in a reaction buffer consisting of 50 mM sodium phosphate buffer pH 7.0, 5 mM EDTA, and 0.005% Tween 20 at 25 °C for 20 min. Standard deviations were calculated from three independent experiments. The fluorescence intensities of the released AMC, ACA, and ACMS were measured with excitation at 355, 350, and 365 nm, respectively, and emission at 460, 450, and 470 nm, respectively, using an Infinite 200 PRO microplate reader (Tecan, Switzerland).

### Enzymatic Reaction in WODLs

A 50 mM sodium phosphate buffer containing 5 mM EDTA was used as a reaction buffer; 50 nM PmDAPBII was used for 100 μM Met–Leu–ACA hydrolysis; and 5 nM PgDPP11 was used for 100 μM Leu–Asp–ACA hydrolysis. These reaction solutions were encapsulated as WODLs using the On-Chip droplet generator. Enzyme reactions were performed at room temperature.

### WODL Cultivation

The bacterial culture medium and *tofu* factory waste fluid were centrifuged at 6000× *g*, and the pellet was washed in 0.9% NaCl solution before suspension in the casitone medium. In both *E. coli* and *P. mexicana*, stochastic encapsulation of microorganisms followed a Poisson distribution^31^ due to the previously investigated relationship between OD_600_ and a colony forming unit (Figure S3). Because the number of bacterial cells in the *tofu* factory waste fluid was not determined, bacterial suspension in the casitone medium was directly used for WODL generation. WODLs containing microorganism cells and a 100 μM ACA substrate were statically cultivated at 30 °C for 1 day.

### Sequencing Analysis

The 16 s rDNA library preparation, sequencing, and data analysis were carried out based on a previous report.^32^ Sequencing methods are described in detail in the Supporting Information. Metagenomic sequencing data were analyzed with the Quantitative Insights Into Microbial Ecology software (version 1.9.1).^33^ Operational taxonomic units (OTUs) were selected at 97% identity with UCLUST. Taxonomic classification was assigned with the Basic Local Alignment Search Tool (BLAST) based on the Greengenes database, version 13_8. The number of reads after quality filtering and OTUs is shown in Table S1. BLAST in the NCBI database (http://blast.ncbi.nlm.nih.gov/Blast.cgi) was used to search for bacterial species corresponding to the 16S rDNA sequence determined by Sanger method sequencing. Sanger sequencing was carried out at Eurofins Genomics (Tokyo, Japan). The metagenomic sequencing data in this study may be obtained from the DNA Data Bank of Japan (DDBJ) database under accession number DRA012262.

### Graphical Programs

Histograms and plots of FADS were produced by FlowJo software, version 10.7.1 (Becton, Dickinson & Company, NJ, USA). Chemical structural formulas were depicted by ChemDraw Std, version 14.0.0.117 (PerkinElmer, MA, USA) and MarvinSketch, version 21.13.0 (ChemAxon, Hungary).

## Results and Discussion

### Examination of Leakage of Fluorescent Substances from the WODL

**Figure 1:**
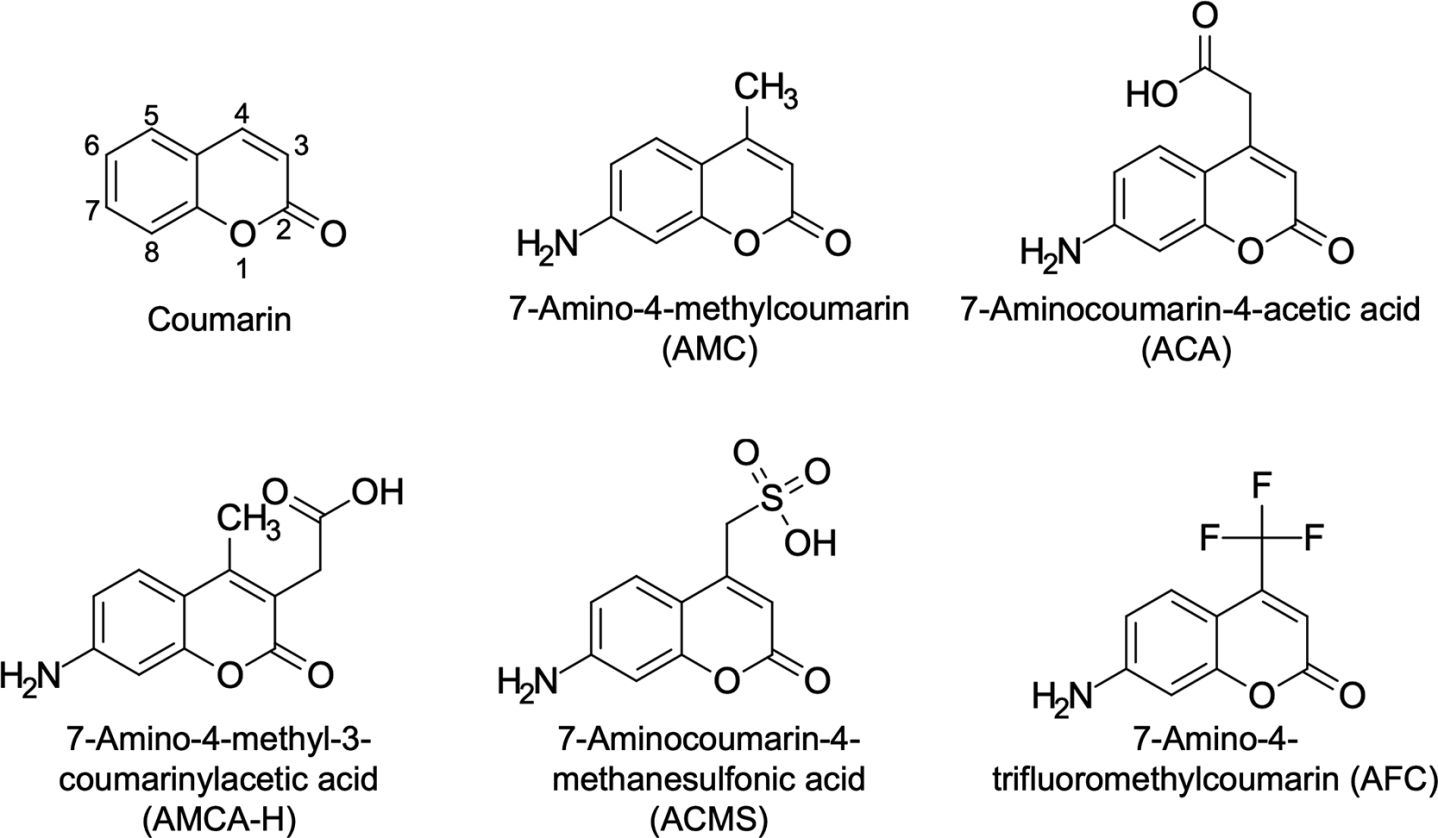
Structural formula of coumarins used in this study.

Hydrophobic fluorescent substances such as AMC leak from WODLs because of the fluorophore exchange between WODLs.^16−18^ Here, we evaluated four coumarin-derived fluorescent substances, AMC, ACA,^26^ AMCA-H,^34^ and ACMS^17^ to assess their ability to remain in WODLs (Figure 1). A WODL with a fluorescent substance (positive WODL) and one with sulforhodamine B instead of the fluorescent substances (negative WODL) were prepared. Fluorescence intensity was measured after positive and negative WODLs were mixed. Since ACMS was not commercially available, it was synthesized in this study (as shown in the Supporting Information). AMC leaked into the oil immediately after mixing, and blue fluorescence was uniform in all WODLs (Figure S4a). AMCA-H showed no leakage immediately after mixing, but the blue fluorescence increased in the negative (non-AMCA-H) WODLs after 168 h (Figure S4b). Indeed, the signal-to-background (*S*/*B*) ratio of positive (containing AMCA-H) and negative WODLs of blue fluorescence decreased as time passed (Table 1). On the other hand, ACA was retained in the WODL even after 168 h (Figure S4c), and the *S*/*B* ratio had a similar value compared to that seen immediately after mixing. As reported previously,^17^ ACMS remained in the WODL even after 168 h due to increased hydrophilicity (Figure S4d), and it had the highest c ratio.

**Table 1:** *S*/*B* Ratios and MiLog*P* Values of the Fluorescent Substances^a^

fluorescent substance	*S*/*B* ratio of each incubation time				MiLog*P*
	0 h	24 h	72 h	168 h	
AMC	1.01	1.00	1.00	1.01	1.44
AMCA-H	18.8	15.6	11.2	6.57	0.80
ACA	22.8	22.8	26.7	26.4	0.42
ACMS	64.9	68.8	66.2	73.4	–1.95

a The *S*/*B* ratio was calculated by dividing the average value of the blue fluorescence of 10 positive WODLs and that of 10 negative WODLs from micrographs. “0 h” refers to microscopic observation immediately after mixing positive and negative WODLs. Micrographs are shown in Figure S4. Blue fluorescence intensities were analyzed by ImageJ.^30^

To investigate the relationship between hydrophobicity and fluorophore exchange, the log*P* value of each compound was calculated by Molinspiration (Table 1). Incidentally, we sought to evaluate AFC, but 100 μM AFC was insoluble in a 50 mM sodium phosphate buffer (pH 7.0). The log*P* value indicated a correlation between hydrophobicity and leakage from the WODL, in agreement with previous reports.^19^ Compared to ACA (MiLog*P*, 0.42) and AMCA-H (MiLog*P*, 0.80), the retentive properties of fluorescent probes in the WODL appeared to vary between log*P* values of 0.42 and 0.80 (Figure S5). Fluorescent probes may be necessary to establish MiLog*P* values below 0.42 when detecting enzyme activity in the WODL. However, since fluorophore exchange is caused by transport with surfactant instead of diffusion,^18^ the log*P* value alone would not be enough to determine whether the fluorescent substance is retained in the WODL rigorously or not. Moreover, although AFC (MiLog*P*, 1.89) is insoluble in water, not all compounds with an MiLog*P* value above 1.89 are insoluble. Indeed, it is possible to dissolve fluorescein (MiLog*P*, 2.56) and resorufin (MiLog*P*, 2.14) in organic solvents diluted with water and encapsulated in the WODL.^16,18,20^ Thus, it is necessary to consider not only the hydrophobicity but also the polarity and the molecular weight of compounds.

### Synthesis of Fluorogenic Substrates and Evaluation of the Detection of Purified DPP Activities in Bulk

Since ACMS and ACA were retained in the WODL, they were considered as candidates for a fluorescent probe to detect DPP activities in WODLs. Toward this, we first synthesized Met–Leu–ACMS and Met–Leu–ACA, both of which would be degradable by bacterial DPP7. It should be noted that the dipeptidyl ACA substrate is a novel substrate that has not been reported yet. Fluorogenic substrates that contain ACA were synthesized using solid-phase peptide synthesis (Figure S6). The Fmoc group was introduced into ACA using Fmoc-Cl, as reported by Harris et al.^26^ The Fmoc-ACA was loaded onto 2-chlorotrityl chloride resin, and the Fmoc group was removed with 20% piperidine. The first Fmoc amino acid was coupled using 1-[bis(dimethylamino)methylene]-1*H*-1,2,3-triazolo[4,5-*b*]pyridinium 3-oxide hexafluorophosphate with collidine, and after deprotection of the Fmoc, the second amino acid was incorporated using *N,N*′-diisopropylcarbodiimide with 1-hydroxybenzotriazole. After the deprotection, the extended peptides were cleaved with a trifluoroacetic acid cocktail. Reverse-phase high-performance liquid chromatography (HPLC) purification was performed to obtain the dipeptidyl ACA substrates. Their synthetic yields were low presumably because of the difficulty in coupling with the first amino acid and the amino group of ACA loaded on the resin. On the other hand, the substrate containing ACMS^17^ was synthesized as shown in Figure S7. Leucine methyl ester was coupled with Boc-methionine, and the subsequent saponification yielded the dipeptide intermediate. We chose a mixed anhydride method for coupling with ACMS. The final deprotection of the Boc group and reverse-phase HPLC purification yielded the ACMS substrate. The synthesis methods are described in detail in the Supplementary Information.

Synthesized Met–Leu–ACMS and Met–Leu–ACA were evaluated to establish whether they could detect purified DPP activity in bulk (noncompartment) and compared with the AMC substrate. PmDAPBII (currently known as bacterial DPP7) and SmDPP7, which are typical bacterial DPP7s showing substrate specificity for Met–Leu–AMC, were used for demonstration purposes.^25,28^ We found that the dipeptidyl ACMS substrate was completely undetectable for DPP activities (Figure S8). For Met–Leu–ACA, this substrate was able to detect for DPP activities. The specificity constant (*k*_cat_/*K*_m_) of DPP7s against this substrate increased approximately fourfold, which is attributed to a lower *K*_m_ value, compared to that of Met–Leu–AMC (Table 2). Therefore, we synthesized Leu–Asp–ACA and examined whether it could detect DPP11 activity, along with DPP7. SmDPP11 and PgDPP11, which possess substrate specificity against Leu–Asp–AMC, were used for verification.^28,35^ DPP activities were successfully detected using Leu–Asp–ACA (Table 2). For SmDPP11, the value of *k*_cat_/*K*_m_ increased compared to that of the Leu–Asp–AMC substrate, similar to DPP7s, but not in PgDPP11. It is noteworthy that the specificity constants increased for three of the four family S46 DPPs measured in this study. In addition, the slight increase in Stokes shift due to change in the maximum fluorescence wavelength to the long wavelength side suggests that ACA is a fluorescent group with a better signal-to-noise ratio than AMC (Figure S1). Therefore, we note that ACA could become a standard fluorescent substance for DPPs replacing AMC.

**Table 2:** Kinetic Parameters of the Bacterial DPP7 and DPP11 toward Synthetic Substrates Measured in Bulk^a^

enzymes	substrates	*V*_max_ (IU mg^–1^)	*K*_m_ (μM)	*k*_cat_ (s^–1^)	*k*_cat_/*K*_m_ (μM^–1^ s^–1^)	ratio of *k*_cat_/*K*_m_ to AMC
PmDAPBII	Met–Leu–AMC	0.637 ± 0.012	5.30 ± 0.12	0.811 ± 0.016	0.153 ± 0.004	1.00
	Met–Leu–ACA	0.483 ± 0.002	1.11 ± 0.02	0.614 ± 0.003	0.552 ± 0.005	3.60
SmDPP7	Met–Leu–AMC	0.672 ± 0.006	21.1 ± 0.5	0.849 ± 0.007	0.0402 ± 0.0007	1.00
	Met–Leu–ACA	0.512 ± 0.003	4.09 ± 0.13	0.647 ± 0.004	0.158 ± 0.004	3.94
PgDPP11	Leu–Asp–AMC	3.93 ± 0.06	10.6 ± 0.2	5.22 ± 0.08	0.492 ± 0.007	1.00
	Leu–Asp–ACA	2.94 ± 0.07	10.1 ± 0.5	3.90 ± 0.10	0.386 ± 0.008	0.785
SmDPP11	Leu–Asp–AMC	14.3 ± 0.4	45.0 ± 1.9	18.0 ± 0.5	0.401 ± 0.007	1.00
	Leu–Asp–ACA	18.1 ± 0.3	12.2 ± 0.4	22.8 ± 0.3	1.88 ± 0.03	4.68

a Standard deviations were obtained from three independent experiments. Michaelis–Menten plots are shown in Figure S8.

Improving the substrate hydrophilicity by adding the sulfo group is an effective way to perform enzyme assay in a WODL. This is supported by the report of Woronoff et al., who succeeded in synthesizing phenylacetyl–ACMS and detecting the activity of *E. coli*-derived acylase in the WODL.^17^ Moreover, phosphotriesterase and cellulase activities could be detected in WODLs by synthesizing a substrate with a sulfo group attached.^19,36^ However, this study revealed that the functional group of the fluorescent substance affects the enzyme reaction rate (Table 2). Although family S46 peptidases such as PmDAP BII do not recognize an amino acid side chain at the prime side subsite^25^ and have a wide space on the prime side (Figure S10), the dipeptidyl ACMS substrate was undetectable for DPP activities. It is predicted that a hydrophobic surface of S46 peptidases would reject a substrate with its high polarity at the entrance of the substrate-binding pocket. This indicated that excessive polarization, as in adding the sulfo group to the fluorescent probe, could negatively affect not only DPPs but also the enzyme activity. On the other hand, ACA binding a carboxyl group improves hydrophilicity and has no negative effect on detecting DPP activity. The present study suggests that adding the sulfo group to the fluorescent probe is not the best way to improve the substrate hydrophilicity for use in the WODL. The functional group should be selected according to the target enzymes.

### Detection of Purified DPP Activities in the WODL

**Figure 2:**
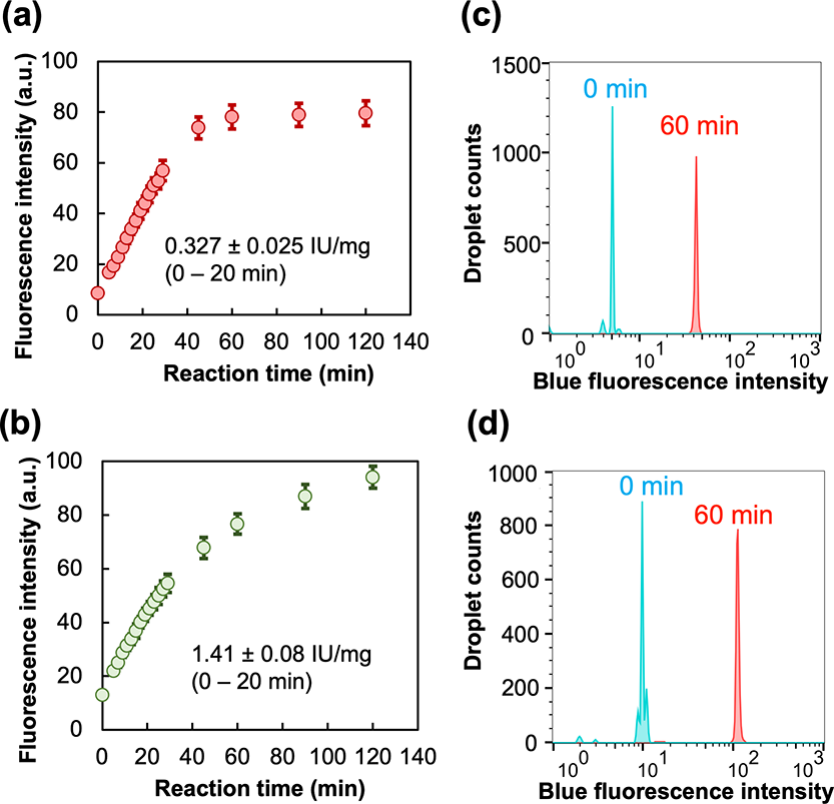
Detection of purified DPP activity in WODLs using dipeptidyl ACA substrates. (a,c) PmDAPBII-hydrolyzed Met–Leu–ACA. (b,d) PgDPP11-hydrolyzed Leu–Asp–ACA. (a,b) Enzyme reaction curves. Fluorescence intensity was measured from microscopy images with ImageJ.^30^ Micrographs used in the analysis are shown in Figure S9. Standard deviations were obtained from the fluorescence intensity of 10 droplet images. (c,d) FADS histogram. “0 min” means analyzing immediately after generating the WODLs. A total of about 10,000 droplets were analyzed by FADS.

The dipeptidyl fluorogenic substrates Met–Leu–ACA and Leu–Asp–ACA were capable of detecting dipeptidyl peptidase activity in bulk (noncompartment). Subsequently, we verified whether the detection of purified enzyme activity was possible in the WODL (compartment). We used 50 nM PmDAPBII and 5 nM PgDPP11 as degradable enzymes for Met–Leu–ACA and Leu–Asp–ACA, respectively. An increase in blue fluorescence intensity was confirmed to be time-dependent in both ACA substrates using microscopy (Figure 2a,b). The specific activities of PmDAPBII and PgDPP11 against Met–Leu–ACA and Leu–Asp–ACA were 0.327 and 1.41 IU/mg, respectively. The lower specific activity than that measured by the bulk method was at a lower temperature than in the bulk method (25 °C) because the temperature was not controlled on the microscope stage. Moreover, the WODLs at zero and 60 min after the reaction were analyzed using FADS, and an increase in blue fluorescence intensity was detected in the WODL at 60 min after the reaction (Figure 2c,d). This suggests that rapid screening using FADS based on DPP activity is possible when using the ACA substrate. To the best of our knowledge, the dipeptidyl ACA substrate is the first fluorogenic substrate to be shown as capable of detecting and quantifying DPP activity in a WODL.

The development of substrates detectable in the WODL enables high-throughput screening and leads to a reduction in the amount of substances used and also facilitates detection of activity with high sensitivity on activity-based screening: 0.5 nM PgDPP11, which shows an activity of 2.94 IU/mg against Leu–Asp–ACA (in bulk assay), was detectable in a WODL by using 100 μM Leu–Asp–ACA. The number of molecules that a 5 nM enzyme encapsulated in a 1 nL droplet was about 5 × 10^–18^ mol (1.17 × 10^–15^ IU for Leu–Asp–ACA). This indicates that a tiny amount of DPP would be detected with high sensitivity using the ACA substrate and WODL. Furthermore, because a 100 μM substrate solution is a tiny amount in a droplet of about 1 nL, a combination of an ACA substrate and a WODL becomes a powerful tool for microorganism screening that requires a large number of samples (e.g., one million samples), reducing the amount of substances used.

### Detection of DPP Activities of Bacterial Cells in a WODL and Screening by FADS

**Figure 3:**
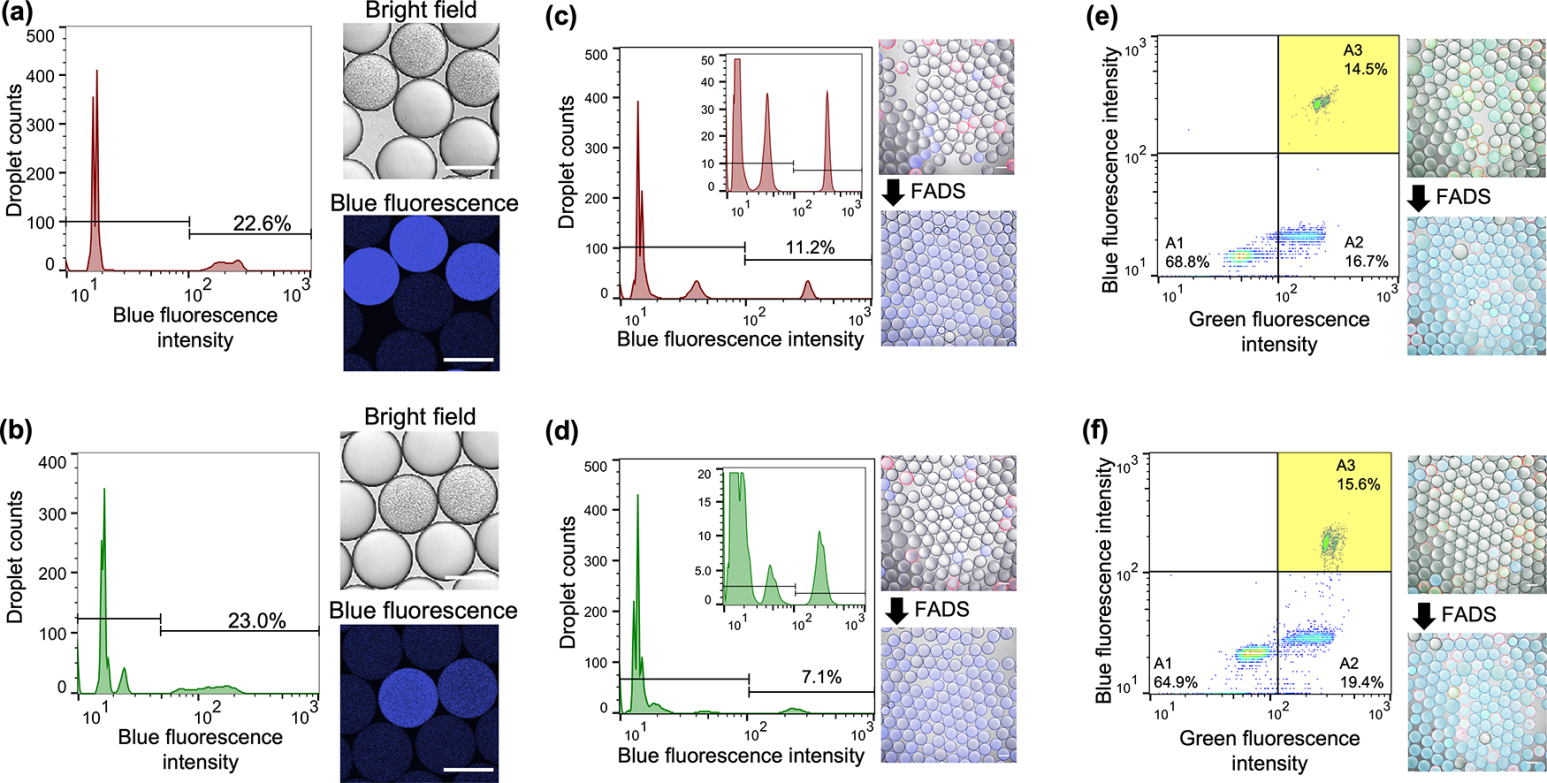
High-throughput isolation of model microorganisms using (a,c,e) Met–Leu–ACA and (b,d,f) Leu–Asp–ACA. (a,b) Detection of DPP activity of the bacterial cell (*P. mexicana* WO24) in WODLs. A total of about 3000 droplets were analyzed by FADS. (b,c) High-throughput sorting of model microorganisms, *P. mexicana* WO24 (DPP-producing) and *E. coli* (DPP nonproducing). *E. coli* expressed RFP. A total of about 3000 droplets were analyzed by FADS. Micrographs before merging are shown in Figure S13. (e,f) High-throughput isolation based on DPP activity from suspensions containing two kinds of bacteria, *P. mexicana* WO24 and *E. coli*. In the pseudocolor plot of FADS, blue and green correspond to areas of lower cell density, red and orange correspond to areas of high cell density, and yellow corresponds to the mid-range. A total of about 8000 droplets were analyzed by FADS. Micrographs before merging are shown in Figure S14. All scale bars in micrographs represent 100 μm.

Here, we validated that rapid screening of microorganisms based on DPP activity using the WODL is possible. Incidentally, ACA substrates were detectable for DPP activities in the casitone medium, including many peptides and amino acids from casein (Figure S11). As a model microorganism, a DPP-producing bacterium *P. mexicana* WO24^27^ and a DPP-nonproducing bacterium *E. coli* DH5α were used. In addition, *E. coli* was transfected with a plasmid containing an RFP coding gene to distinguish between *E. coli* and *P. mexicana* WO24. Both microorganisms (not mixture) were each compartmentalized into WODLs along with ACA substrates and the casitone medium. For *P. mexicana* WO24, an increase in blue fluorescence showing DPP activity with the growth of microorganisms was observed using both Met–Leu–ACA and Leu–Asp–ACA substrates (Figure 3a,b). The WODLs with increased blue fluorescence could be separated by FADS. For *E. coli*, although red fluorescence associated with their growth was detected, blue fluorescence was not increased with either of the ACA substrates (Figure S12). We attempted to separate *P. mexicana*- and *E. coli*-encapsulated WODLs based on DPP activity. FADS sorted 11.2% (with Met–Leu–ACA) and 7.1% (with Leu–Asp–ACA) of the WODLs’ elevated blue fluorescence values due to degradation of the ACA substrates (Figure 3c,d). Microscopic analysis showed that only *P. mexicana* WO24-encapsulated WODLs were isolated according to DPP activity using FADS on each substrate. These results demonstrate that DPP-producing microorganisms could be rapidly screened by FADS using the dipeptidyl ACA substrates.

One of the advantages of the WODL for microorganism screening is compartmentalization. Thus, *E. coli* and *P. mexicana* were mixed before formation of WODLs, and only the *P. mexicana*-encapsulated WODL was isolated from the two kinds of bacteria mixed in solution based on DPP activity. In addition, FNAP-sort was included in the WODLs to distinguish the WODLs in which bacteria grew.^15^ FNAP-sort is a fluorescent nucleic acid substrate that emits green fluorescence when degraded by RNase produced by microorganisms. Figure 3e,f shows the result of FADS after cultivation at 30 °C for 1 day. The WODLs were divided into three areas by green and blue fluorescence intensities as follows: (A1) this area with low green and blue fluorescence showed empty WODLs with no microorganisms and no DPP activity; (A2) this area with high green fluorescence and low blue fluorescence showed *E. coli*-encapsulated WODLs, which grew the DPP nonproductive microorganism; and (A3) this area with high green and blue fluorescence showed *P. mexicana*-encapsulated WODLs because microorganisms grew and produced DPP. In practice, only the *P. mexicana*-encapsulated WODLs could be isolated after WODLs distributed in A3 were sorted. However, RFP fluorescence derived from *E. coli* was detected in *P. mexicana*-encapsulated WODLs. This is because both *P. mexicana* and *E. coli* are simultaneously encapsulated with a certain probability when a WODL is encapsulated according to Poisson distribution. In this connection, the abundance ratio of each WODL follows approximately equal numbers of the Poisson distribution (Figure S3). Consequently, high throughput isolation of a DPP-producing bacterium based on DPP activity from a microorganism mixture is possible by using a dipeptidyl ACA substrate and WODL.

### Isolation of DPP-Producing Bacteria from the Environment

**Figure 4:**
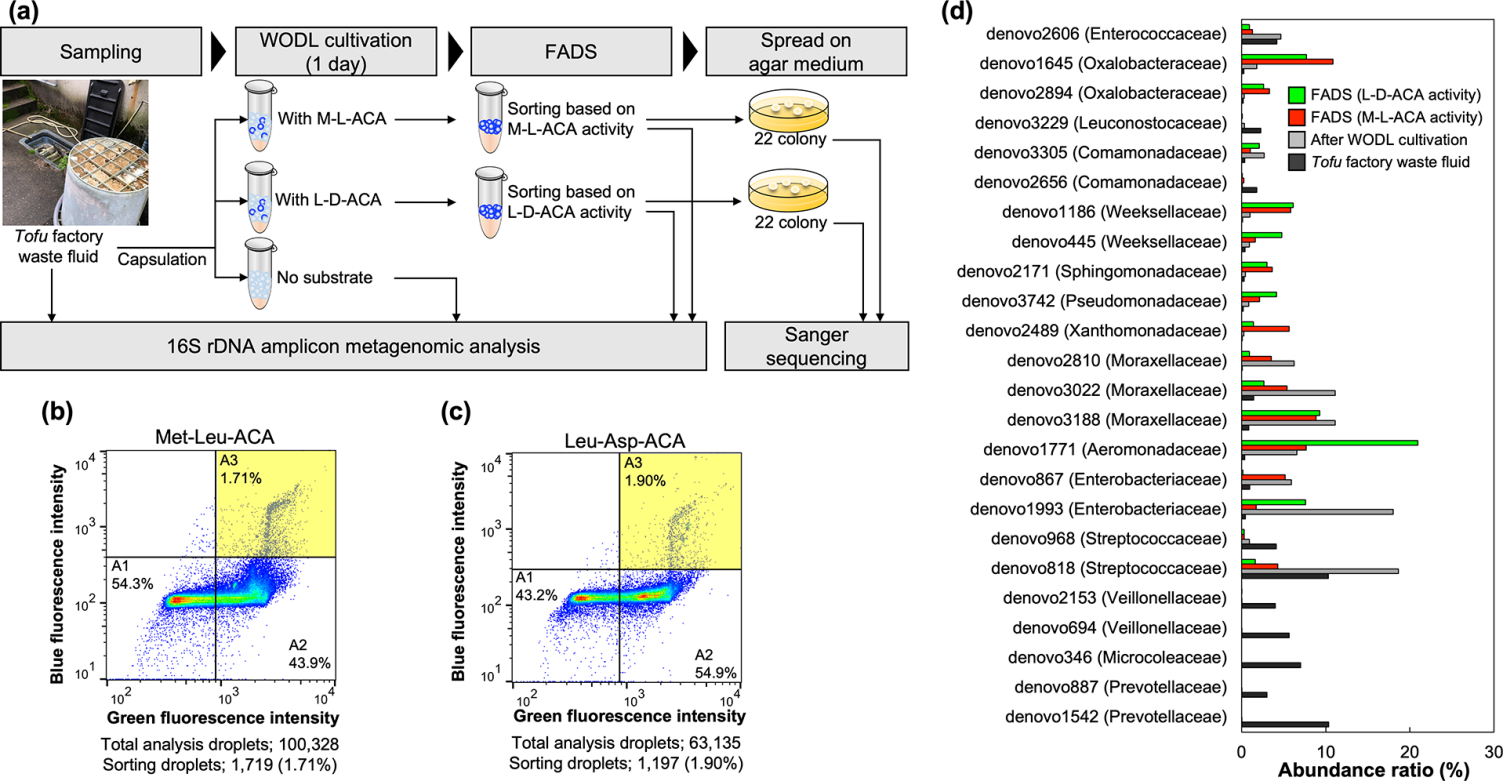
High-throughput screening of environmental microorganisms based on DPP activity. (a) Scheme overview of application of the dipeptidyl ACA substrate for high-throughput screening of DPP-producing bacteria. (b) and (c) Pseudocolor plot of FADS for high-throughput WODLs, sorted using (b) Met–Leu–ACA and (c) Leu–Asp–ACA. Blue and green correspond to areas of lower cell density, red and orange are areas of high cell density, and yellow is mid-range. (d) Abundance ratio of the top 10 OTUs of each sample. FADS (M–L–ACA activity) and FADS (L–D–ACA activity) refer to postsorting samples based on Met–Leu–ACA and Leu–Asp–ACA hydrolysis activity, respectively. The name in parentheses is the family to which each OTU belongs. The abundance ratio was calculated based on 16S rDNA sequence data performed with the MiSeq System (shown in the Supporting Information).

To demonstrate high-throughput screening of environmental microorganisms using the ACA substrate, microorganism screening was performed based on DPP activity from *tofu* factory waste fluid, which is expected to be rich in a protein substrate (Figure 4a). A bacterial cell suspension was enclosed in a WODL with each of the ACA substrates and an FNAP-sort. After cultivation at 30 °C for 24 h, some WODLs showed blue fluorescence from DPP activity and green fluorescence from bacterial growth, and the WODLs in the A3 area were sorted at a maximum rate of about 300 droplets/s by FADS (Figure 4b,c). The sorted WODLs were dispersed by surfactant-free fluorine oil, and only the aqueous phase containing the microorganism was spread on the LB agar medium. A total of 22 colonies screened based on the ACA substrate activity were randomly isolated. The species were identified by 16S rRNA gene amplicon sequencing. A total of 30 species were identified in screening using each ACA substrate (Table S2). In particular, *Stenotrophomonas rhizophila*, *Sphingomonas yabuuchiae*, *Pseudoduganella danionis*, *Flavobacterium chilense*, *Chryseobacterium camelliae*, and *Aeromonas hydrophila* were isolated based on both Met–Leu–ACA and Leu–Asp–ACA hydrolysis activity. A homology search was performed using BLAST^37,38^ to determine whether each species carries the DPP genes.^39^ Among the DPP derived from microorganisms, DPP3, DPP5, DPP7, and DPP11, which have the potential to hydrolyze Met–Leu–ACA and/or Leu–Asp–ACA, were selected. In total, 17 strains possessed DPP genes, most of them carrying the S46 peptidases DPP7 and DPP11 (Table S2). This result shows that it is possible to isolate microorganisms that possess DPP from the environment using the dipeptidyl ACA substrate with high throughput.

### Comparative Analysis of 16S rDNA

The abundance of bacteria in the *tofu* factory waste fluid sample, WODL cultivation sample, and postsorting samples was analyzed using a 16S rRNA amplicon metagenomic approach to confirm whether DPP-producing microorganisms are concentrated according to DPP activity screening using the ACA substrate. The abundance ratios of each sample’s top 10 OTUs are shown in Figure 4d. Prevotellaceae, Microcolaceae, and Veillonellaceae were relatively abundant in the *tofu* factory waste fluid sample. However, they were in low abundance after WODL cultivation. In contrast, some microorganisms with low abundance in the *tofu* factory waste fluid sample were increased by WODL cultivation. For instance, the abundance ratios of Moraxellaceae (denovo2810), Enterobacteriaceae (denovo1993), and Aeromonadaceae (denovo1771), whose abundance ratio for the *tofu* factory waste fluid sample was 1% or less, were increased by 93.6-fold, 39.7-fold, and 17.3-fold, respectively, by WODL cultivation.

Comparison of the *tofu* factory waste fluid sample to FADS based on Met–Leu–ACA hydrolysis showed that the concentration ratio of Xanthomonadaceae (denovo2489) was the highest among postsorting samples and was 76.6-fold. The abundance ratios of Moraxellaceae (denovo2810), Weeksellaceae (denovo1186), and Oxalobacteraceae (denovo1645) in the FADS (Met–Leu–ACA) sample were increased by 52.8-fold, 46.4-fold, and 43.8-fold, respectively, compared to the *tofu* factory waste fluid sample. Some species classified as Xanthomonadaceae, Moraxellaceae, Oxalobacteraceae, or Weeksellaceae, which belong to Proteobacteria or Bacteroidetes, possess bacterial DPP7 and DPP11.^24,40,41^ For the FADS (Leu–Asp–ACA) sample, the abundance ratio of Aeromonadaceae (denovo1771) increased the most, by 55.1-fold. The abundance ratios of Weeksellaceae (denovo1186), Oxalobacteraceae (denovo1645), and Pseudomonadaceae (denovo3742) increased 48.7-fold, 31.1-fold, and 23.3-fold, respectively, compared to the *tofu* factory waste fluid sample. Similar to the FADS (Met–Leu–ACA) sample, some species classified as Aeromonadaceae, Oxalobacteraceae, Weeksellaceae, or Pseudomonadaceae belong to the Proteobacteria or Bacteroidetes phylum and possess the S46 peptidases.^24,40,41^ Therefore, these results support that the screening of microorganisms based on DPP activity using the dipeptidyl ACA substrates succeeded in enriching the abundance of microorganisms possibly through bacterial DPP7 or DPP11.

## Conclusions

This is the first study demonstrating that an ACA substrate can be used to detect the enzyme activity of microorganisms in WODLs. Interestingly, the dipeptidyl ACA substrates detect bacterial DPP activity with more sensitivity than AMC substrates, which is attributed to the decrease in the *K*_m_ values. This indicates that ACA is also a suitable fluorescent probe for evaluating bacterial DPP activity in bulk after screening. S46 peptidases, bacterial DPP7s, and DPP11s are important enzymes for bacterial growth and are promising targets for antimicrobial agents.^28^ The dipeptidyl ACA substrates developed in this study are useful for screening antimicrobial compounds targeting S46 peptidases from natural compounds. Since ACA can modify substrates such as peptides, its range of applications will extend to peptidases other than bacterial DPP. This work would be the base for a highly efficient screening platform for detecting bacterial enzyme activity using the WODL and will not be limited to only DPPs.

## Supporting Information Available

Figures and micrographs showing several control experiments for activity measurement using ACA substrates and tables involved in sequencing analysis; methods describing substrate synthesis and DNA sequencing (PDF)
