# Supplementary material for: 7-Aminocoumarin-4-acetic Acid as a Fluorescent Probe for Detecting Bacterial Dipeptidyl Peptidase Activities in Water-in-Oil Droplets and in Bulk
Source: Anal Chem. 2021 Dec 28;94(5):2416–24. doi: 10.1021/acs.analchem.1c04108 (PMC8886566; doi:10.1021/acs.analchem.1c04108)
Supplement: Supplementary file 1 — ac1c04108_si_001.pdf [file ac1c04108_si_001.pdf]

Supporting Information

7-Aminocoumarin-4-acetic acid as a fluorescent probe for detecting bacterial dipeptidyl peptidase activities in water-in-oil droplets, and in bulk.

Akihiro Nakamura,[†] Nobuyuki Honma,[‡] Yuma Tanaka,[‡] Yoshiyuki Suzuki,[†] Yosuke Shida,[‡] Yuko Tsuda,[§] Koushi Hidaka,[¶]* and Wataru Ogasawara.[†‡]*

[†]Department of Science of Technology Innovation, Nagaoka University of Technology, 1603-1 Kamitomioka, Nagaoka, Niigata 940-2188, Japan; [‡]Department of Bioengineering, Nagaoka University of Technology, 1603-1 Kamitomioka, Nagaoka, Niigata 940-2188, Japan; [§]Faculty of Pharmaceutical Sciences, Cooperative Research Center of Life Sciences, Kobe Gakuin University, 1-1-3 Minatojima, Chuo-ku, Kobe, Hyogo 650-8586, Japan; [¶]Graduate School of Health Sciences, Kobe University, 7-10-2 Tomogaoka, Suma-ku, Kobe, Hyogo 654-0142, Japan.

*Correspondence and requests for materials should be addressed to
K.H. (khidaka@people.kobe-u.ac.jp) and W.O. (owataru@vos.nagaokaut.ac.jp)

Supporting information includes:
Figures S1–S15;
Tables S1 and S2;
Supplemental Materials and Methods.

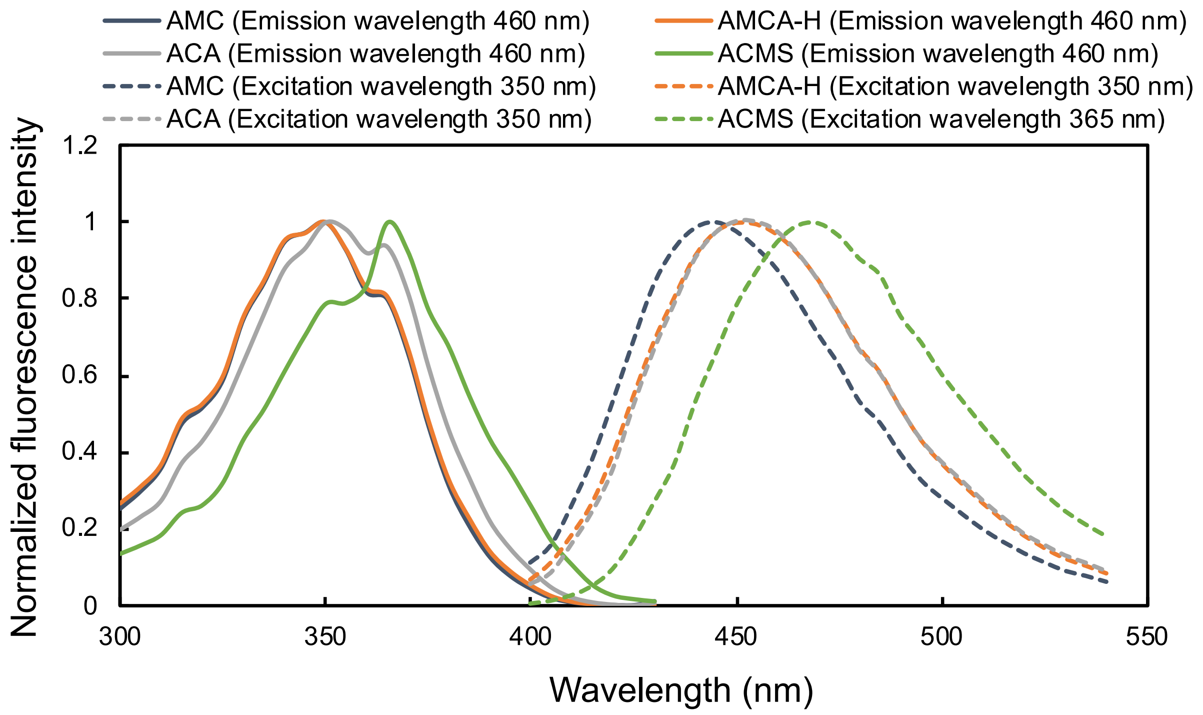

Fluorescent substances	Maximum Ex. (nm)	Maximum Em. (nm)
AMC	350	445
ACA	350	450
ACMS	365	470
AMCA-H	350	450

Figure S1 | Excitation and emission spectra of coumarins used in this study.

The solid and dotted lines represent spectra of excitation light and emission light, respectively. The fluorescence intensity was measured using an Infinite 200 PRO microplate reader (Tecan, Switzerland).

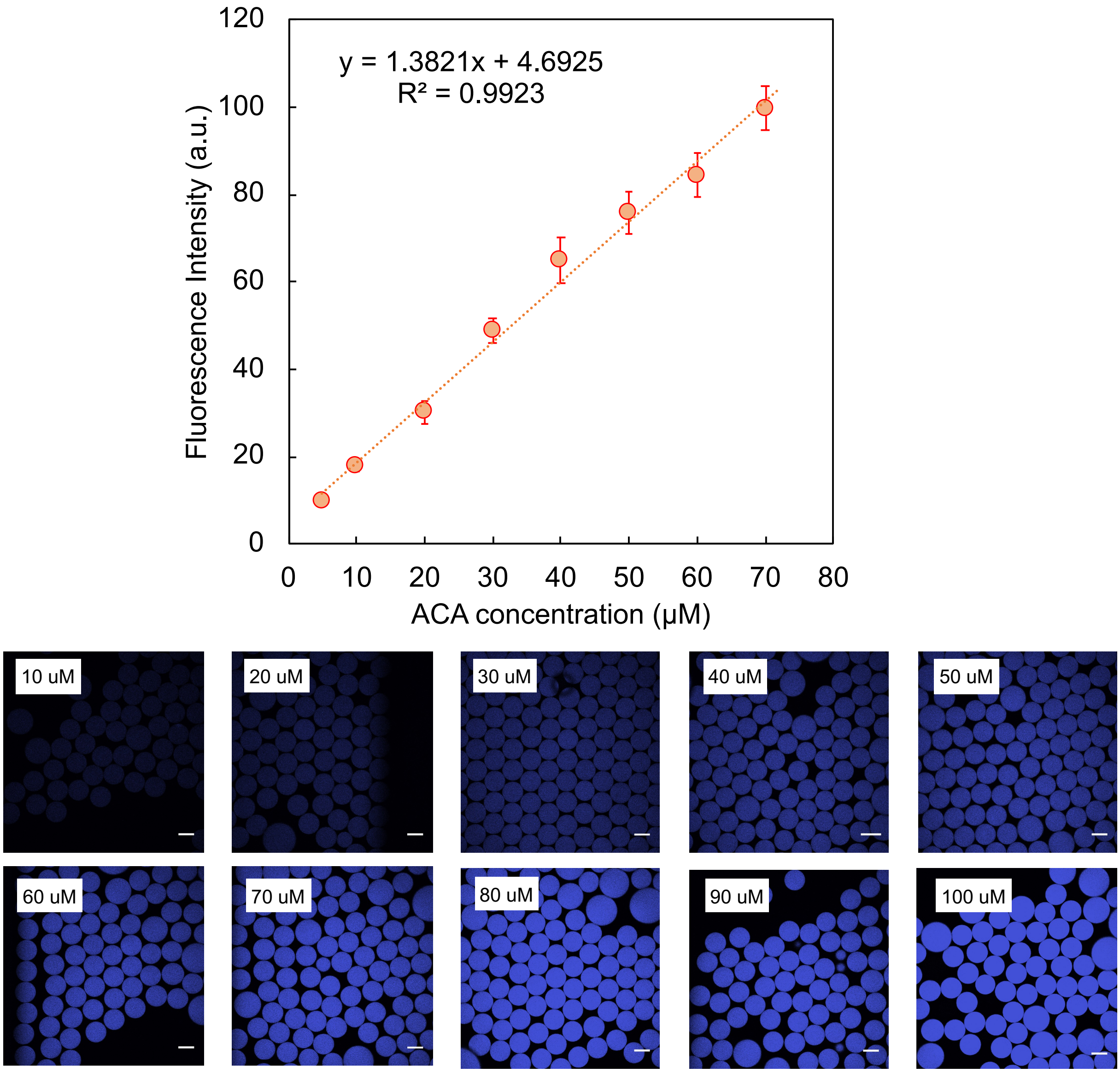

Figure S2 | Calibration curve of ACA concentration in image analysis.
Droplets containing each ACA concentration were prepared, and the fluorescence intensity was analyzed using ImegeJ.[1] Standard deviations were obtained from 20 droplets. The scale bar represents 100 μm.

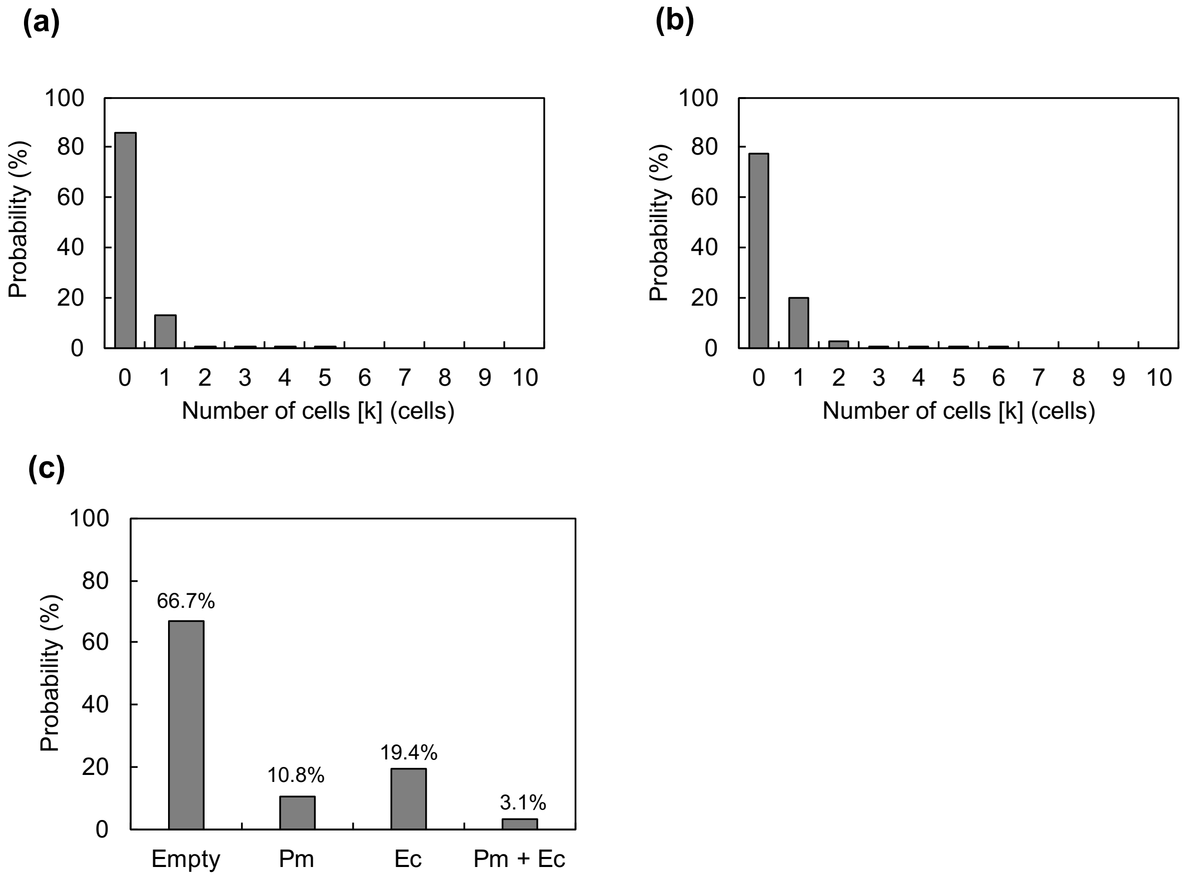

Figure S3 | Poisson distribution[2]

(a) (b) The probability $P(X = x)$ of finding x entities (bacterial cells) was calculated by the equation $P(X = x) = e^{-\lambda}(\lambda^x/x!)$, where e is Napier's constant and λ is the number of bacterial cells in each droplet volume, set at 0.150 for *P. mexicana* WO24 (a) and at 0.255 for *E. coli* (b). (c) The probability of co-encapsulation $P(X_1 = x_1, X_2 = x_2)$ of x_1 cells (*P. mexicana* WO24; Pm) and x_2 cells (*E. coli*; Ec) in a single droplet was calculated by the equation $P(X_1 = x_1, X_2 = x_2) = [e^{-\lambda_1}(\lambda_1^{x_1}/x_1!)] \times [e^{-\lambda_2}(\lambda_2^{x_2}/x_2!)]$. Here, λ was set at 0.125 for *P. mexicana* WO24 and at 0.213 for *E. coli*. 'Empty' refers to an empty droplet with no microorganisms enclosed. 'Pm' is a droplet in which only *P. mexicana* WO24 was enclosed in one or more cells. 'Ec' is a droplet in which only *E. coli* was enclosed in one or more cells. 'Pm + Ec' is a droplet in which *P. mexicana* WO24 and *E. coli* were enclosed in one or more cells of each microorganism.

(a) AMC

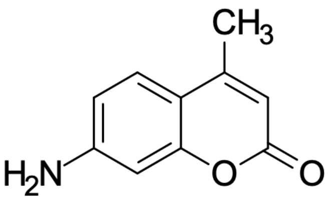
CH3
H2N
O
O

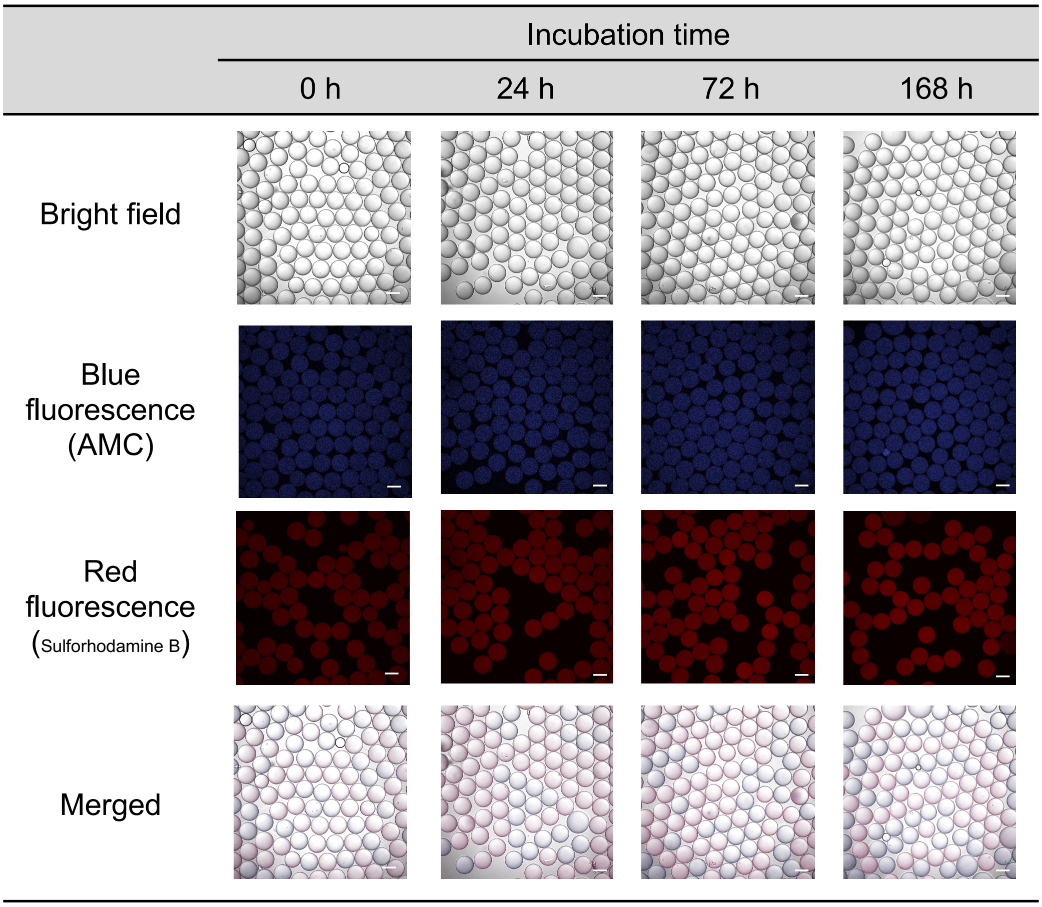
Incubation time
0 h
24 h
72 h
168 h
Bright field
Blue fluorescence (AMC)
Red fluorescence (Sulforhodamine B)
Merged

(b) AMCA-H

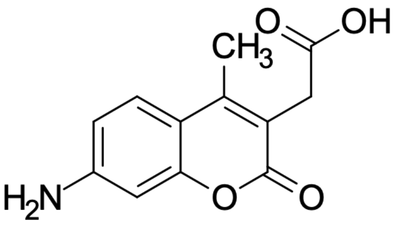
O
OH
CH3
H2N
O
O

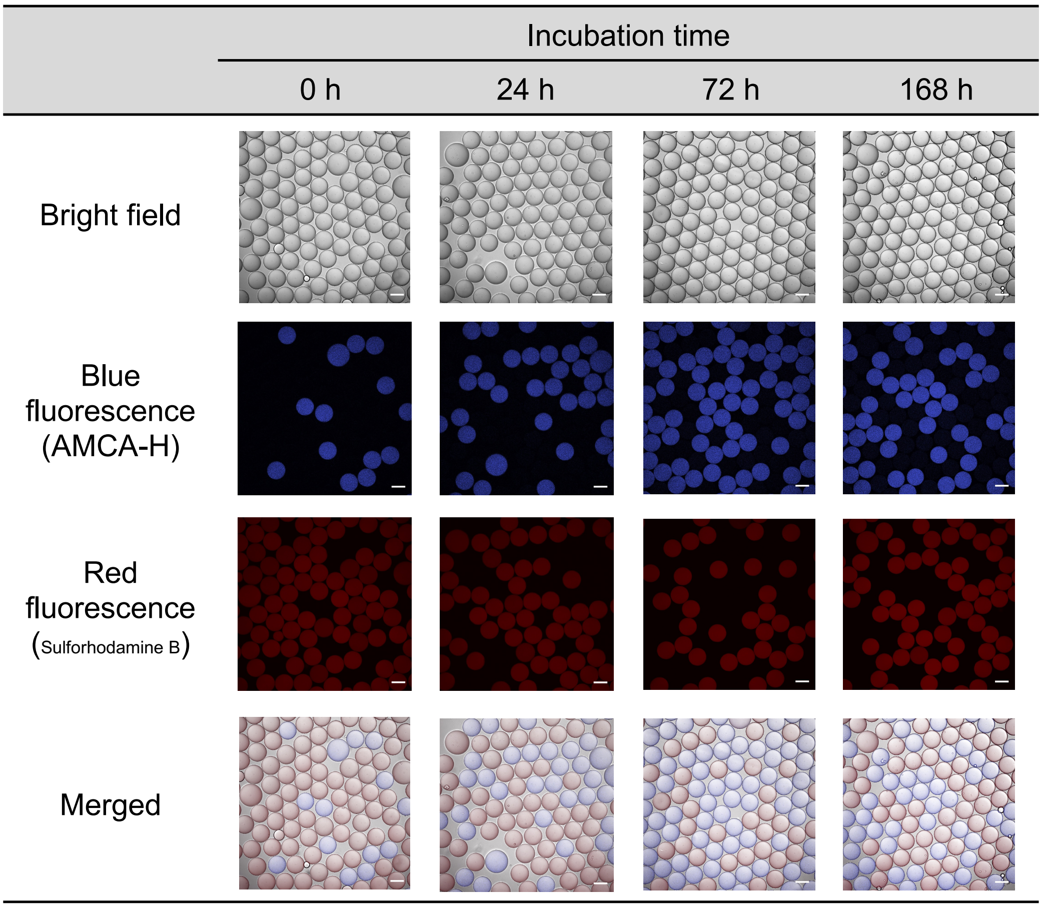
Incubation time
0 h
24 h
72 h
168 h
Bright field
Blue fluorescence (AMCA-H)
Red fluorescence (Sulforhodamine B)
Merged

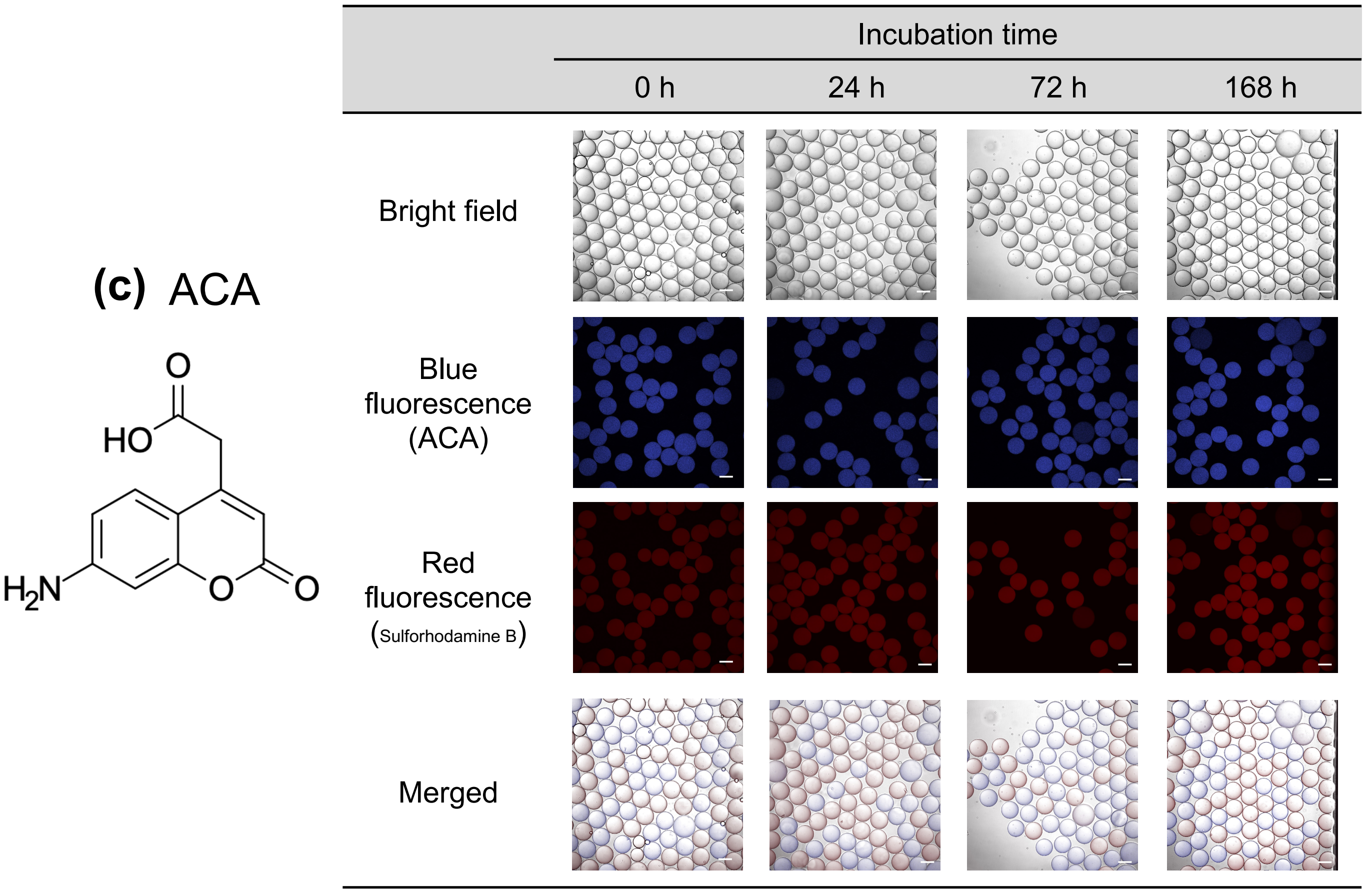
(c) ACA
Incubation time
0 h
24 h
72 h
168 h
Bright field
Blue fluorescence (ACA)
Red fluorescence (Sulforhodamine B)
Merged
HO
O
H2N
O
O

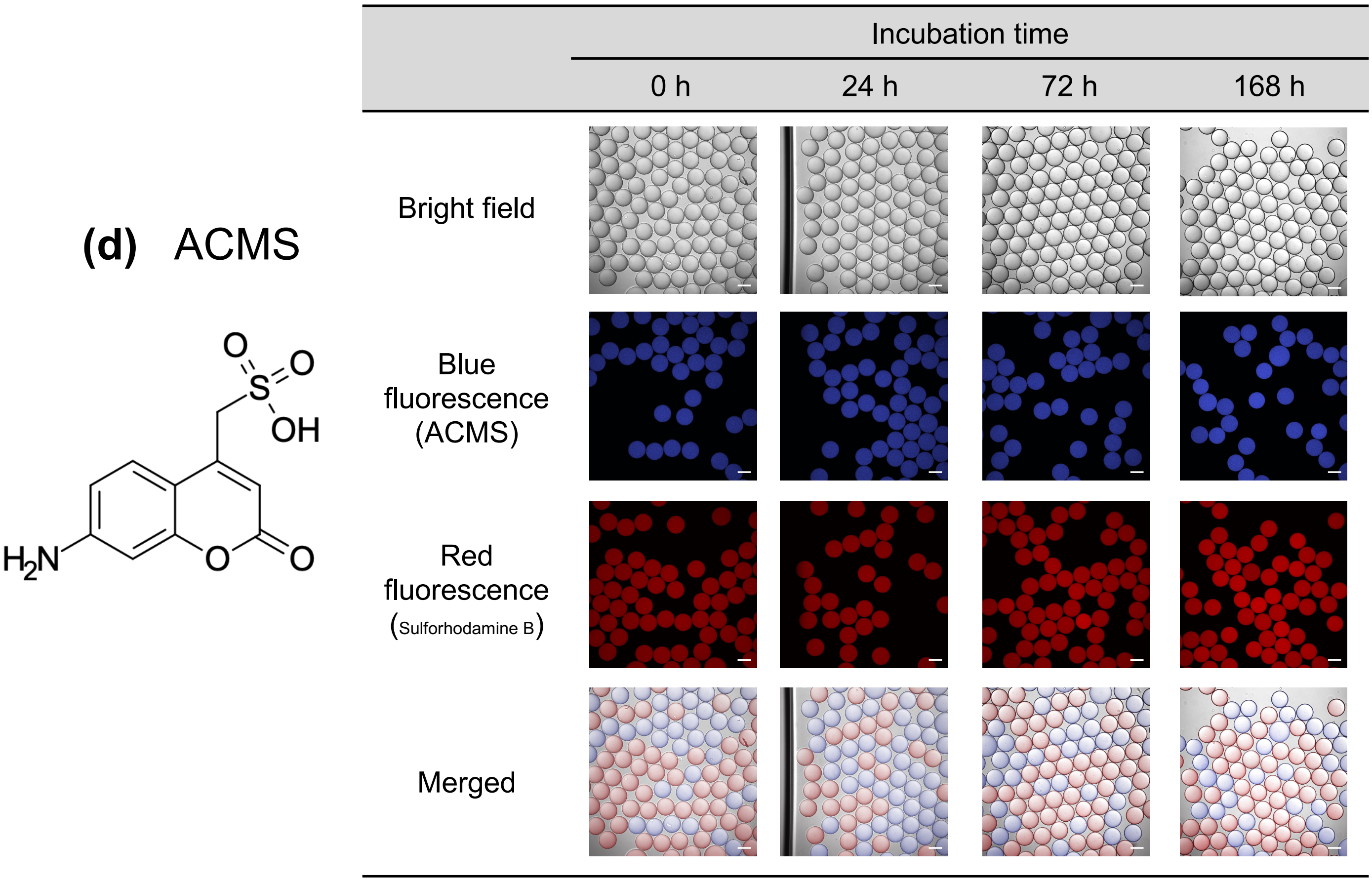
(d) ACMS
Incubation time
0 h
24 h
72 h
168 h
Bright field
Blue fluorescence (ACMS)
Red fluorescence (Sulforhodamine B)
Merged
O
S
O
OH
H2N
O
O

Figure S4 | Leakage examination of the fluorescent compounds.

(a) AMC. (b) AMCA-H. (c) ACA. (d) ACMS. A quantity of 100 μM of each fluorescent substance was encapsulated in fluorescent WODLs and 10 μM of sulforhodamine B was contained in non-fluorescent substance WODLs. 0 h refers to the time immediately after mixing WODLs. Mixed WODLs were incubated at 30 °C for seven days. Micrographs were obtained by the confocal microscope A1 HD25 under 100x magnification. The scale bar represents 100 μm.

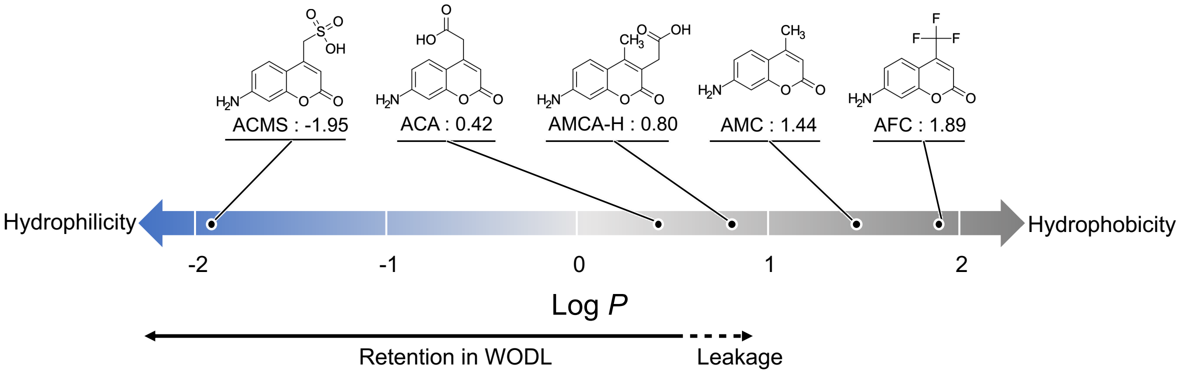

Figure S5 | Schematic diagram of the relationship between log*P* of coumarins used in this study and retentive properties in the WODL.

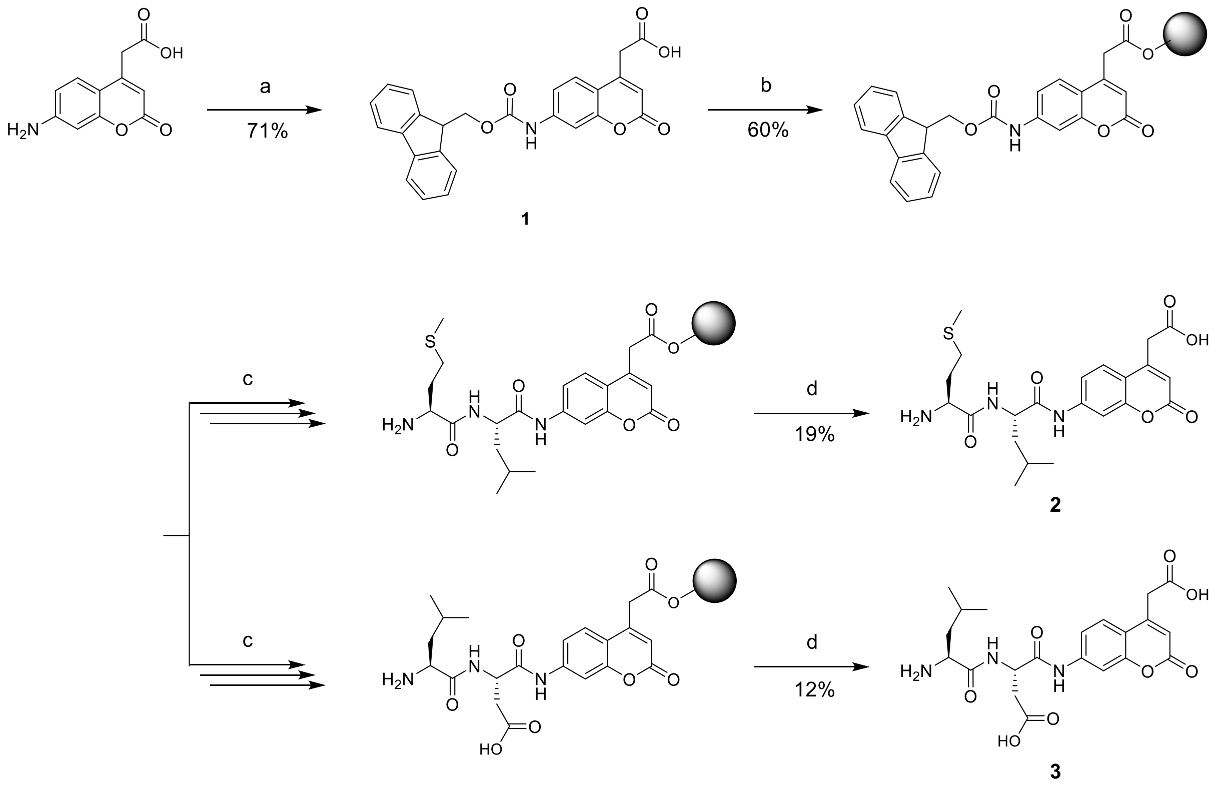

Figure S6 | Synthesis of dipeptidyl-ACA 1 and 2.[a]

[a] (a) Fmoc-Cl, $NaHCO_3$ aq. 18 h; (b) 2-chlorotrityl chloride resin, DIEA, dichloromethane, 17 h; (c) i. 20% peperidine/DMF, 20 min; ii. Fmoc-Leu-OH or Fmoc-Asp(tBu)-OH, HATU, 2,4,6-collidine/DMF, 24 h; iii., 20% peperidine/DMF, 20 min; iv. Fmoc-Met-OH or Fmoc-Leu-OH, DIC, HOBt/DMF, 2h; v. 20% peperidine/DMF, 20 min; (d) i. TFA/TIS/H_2O/DODT (92.5:2.5:2.5:2.5) or TFA/TIS/H_2O (95:2.5:2.5), 2 h; ii. Prep. RP-HPLC purification.

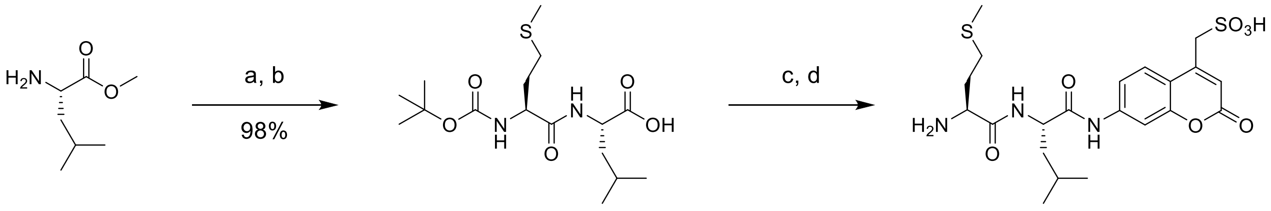

4 **5**

Figure S7 | Synthesis of dipeptidyl-ACMS 5.[a]

[a] (a) Boc-Met-OH, Et_3N, EDC, HOBt/DMF, overnight; (b) 1M NaOH aq., MeOH, 2 h; (c) i. isobutyl chloroformate, 4-methylmorpholine/DMF, 30 min; ii. ACMS, 4-methylmorpholine/DMF, overnight; (d) i. 4M HCl/EtOAc, anisole, 1 h; ii. Prep. RP-HPLC purification.

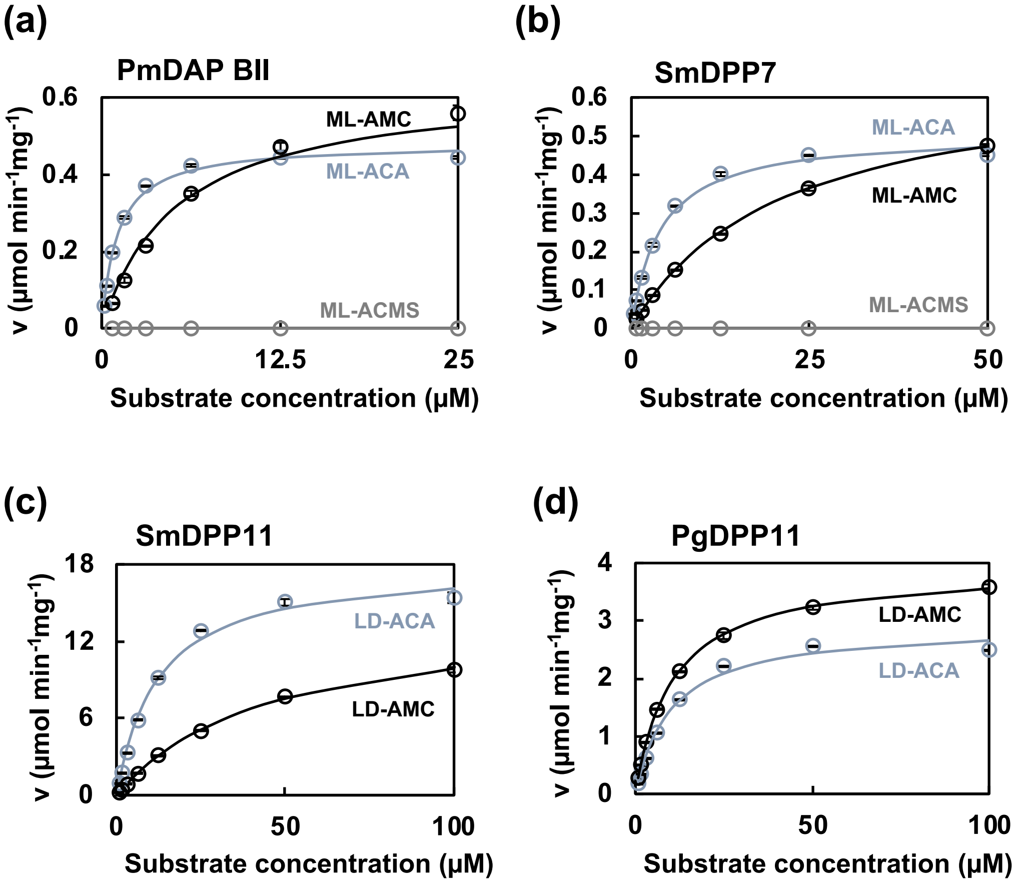

Figure S8 | Michaelis–Menten plots of PmDAP BII (a), SmDPP7 (b), SmDPP11 (c), and PgDPP11 (d).

Enzymatic reaction was performed in a 50 mM sodium phosphate buffer pH 7.0 containing 5 mM EDTA and 0.005% Tween 20 at 25 °C for 20 min. Standard deviations were obtained from three independent experiments.

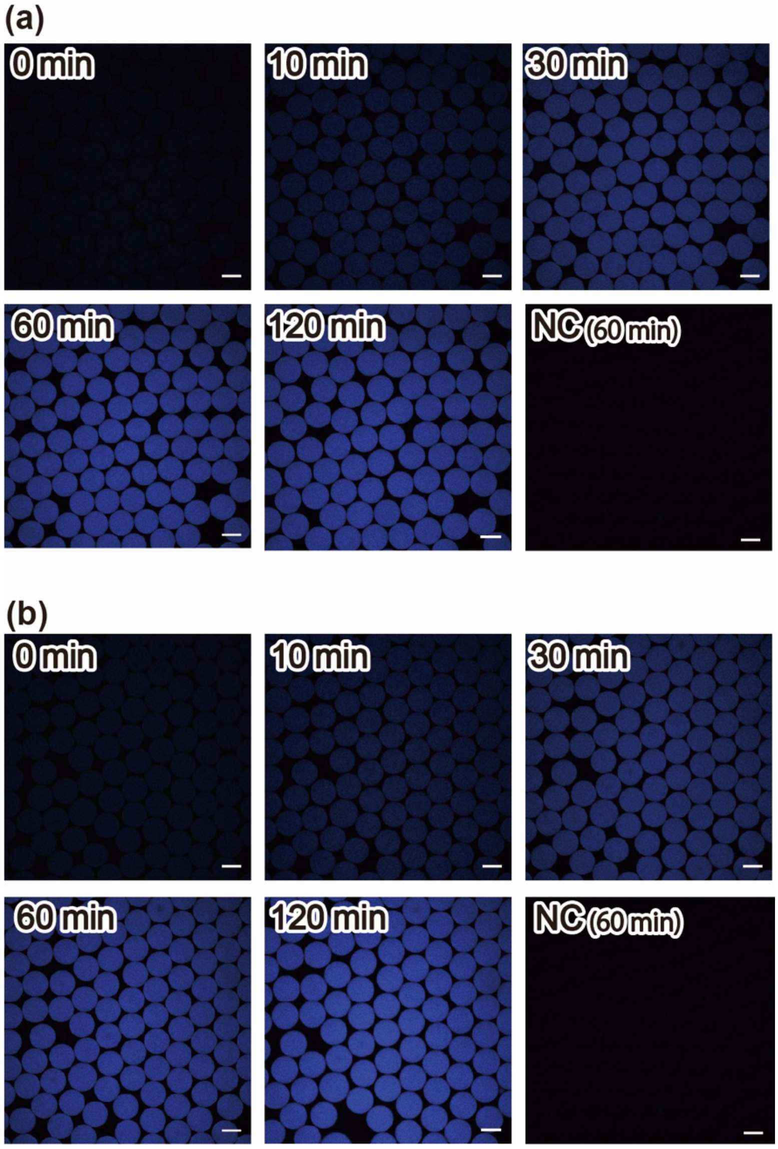

Figure S9 | Detection of DPP activity in WODLs using dipeptidyl ACA substrates.

(a) PmDAPBII hydrolyzed Met-Leu-ACA. (b) PgDPP11 hydrolyzed Leu-Asp-ACA. Enzymatic reaction was performed in a 50 mM sodium phosphate buffer pH 7.0 containing 5 mM EDTA at room temperature in WODL. Micrographs were obtained by a confocal microscope under 100x magnification. 'NC' means no enzyme, only substrate and buffer. The scale bar represents 100 μm.

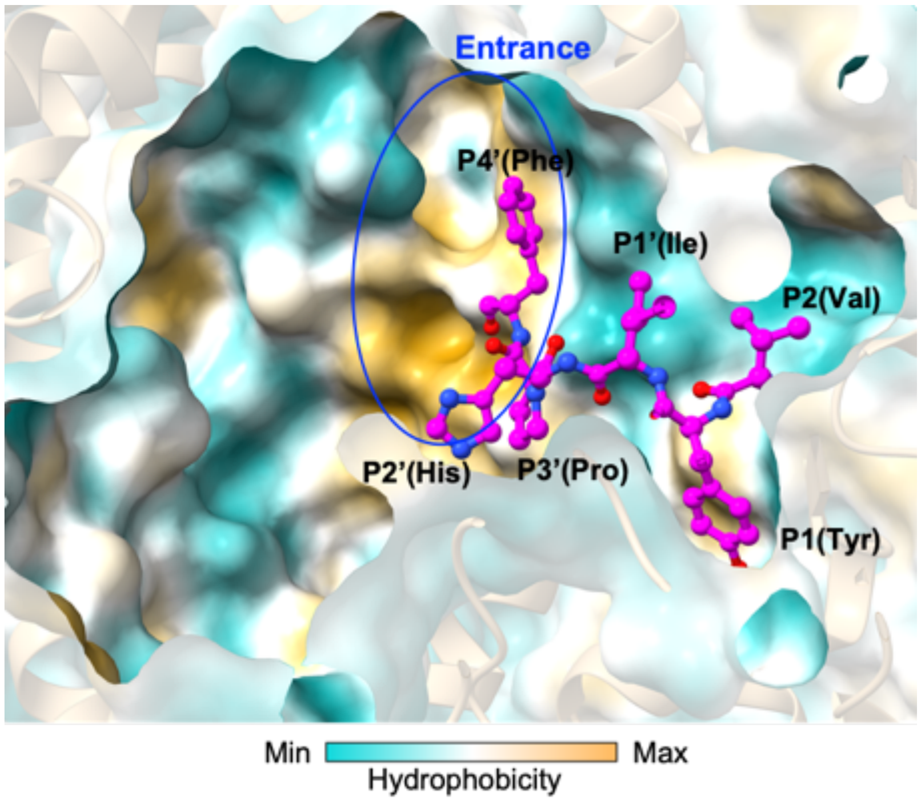

Figure S10 | Structure of the substrate binding pocket with surface hydrophobicity of PmDAP BII (PDB; 3WOQ).

Protein structural model produced using the program UCSF Chimera X.[3] Map values for surface '3woq.pdb_A SES surface': minimum -28.39, mean -5.109, maximum 22.83. MLPP: A program for the calculation of molecular lipophilicity potential in proteins.[4]

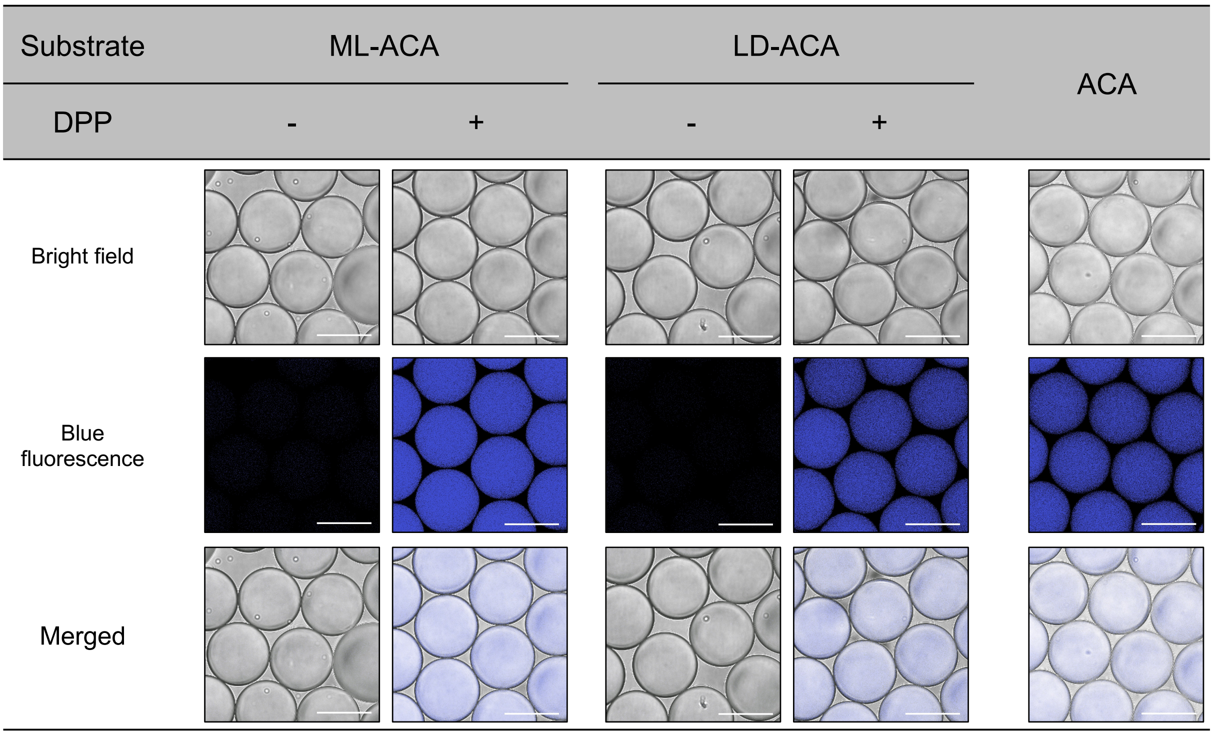

Figure S11 | Detection of DPP activity using ACA substrate in casitone medium. Micrographs were obtained by the confocal microscope A1 HD25 under 200× magnification. The scale bar represents 100 µm. Blue fluorescence shows DPP activity. For Met-Leu-ACA, 1 nM PmDAPBII and 20 µM Met-Leu-ACA were mixed in casitone medium. For Leu-Asp-ACA, 1 nM PgDPP11 and 20 µM Leu-Asp-ACA were combined in casitone medium. The control was 20 µM ACA in casitone medium. These reaction solutions were encapsulated as WODLs using HFE-7500 3M Novec Engineered fluid (HFE-7500) with a fluorine oil containing 2% (w/w) 008-FluoroSurfactant (RAN Biotechnologies, MA, USA). Enzyme reaction was performed at 30 °C for 24 h in the WODL.

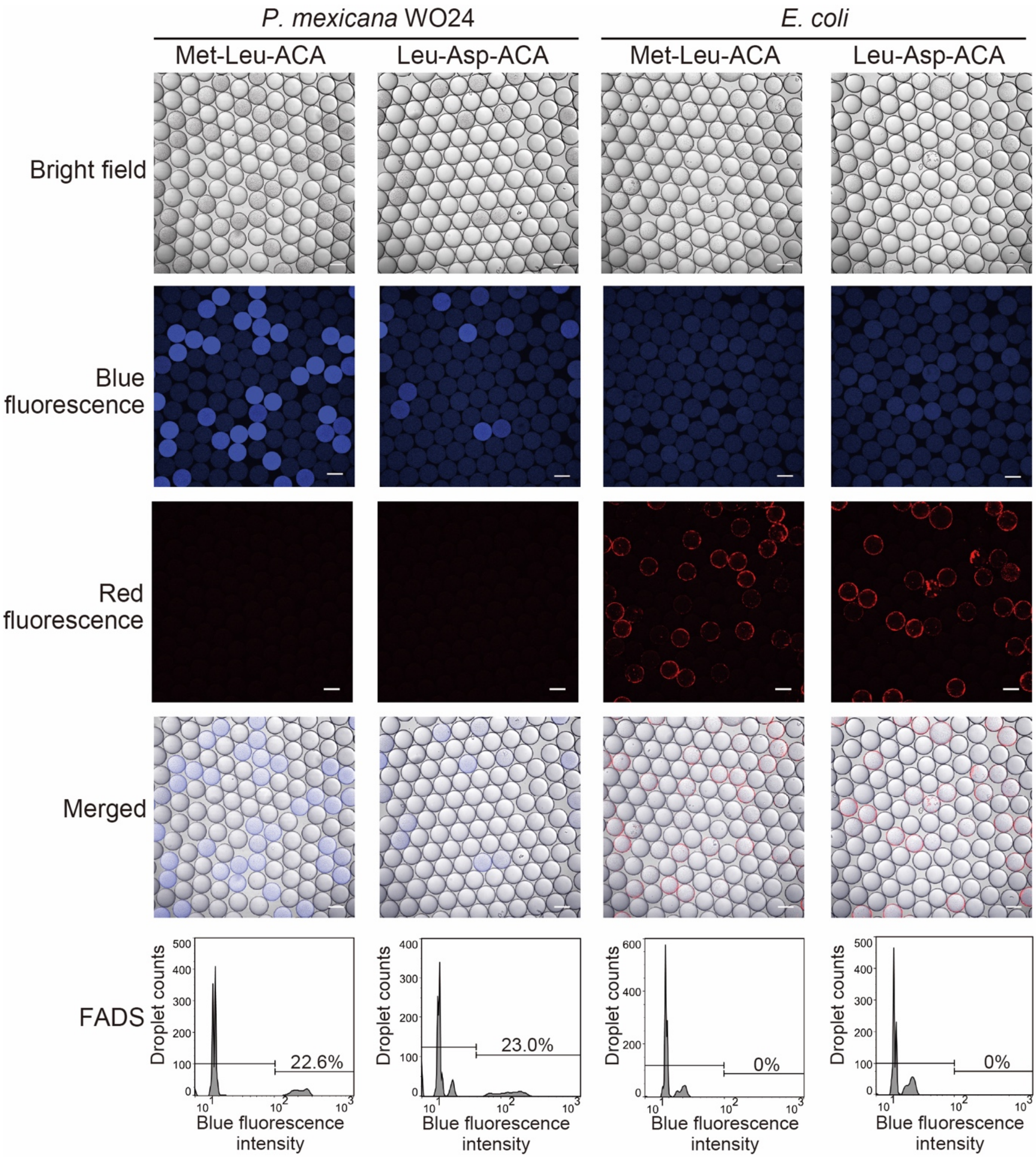

Figure S12 | Detection of DPP-producing and non-producing bacteria in WODLs using dipeptidyl ACA substrates.

P. mexicana WO24 and *E. coli* were used as model microorganisms for DPP-producing and non-producing bacteria, respectively. Each microorganism was encapsulated in WODLs together with ACA substrates and cultured for 24 hours at 30 °C. *E. coli* was transfected with a plasmid containing an RFP-coding gene. FADS was performed using On-Chip Sort, and a total of about 3,000 droplets were analyzed. Micrographs were obtained by the confocal microscope A1 HD25 under 100× magnification. Blue fluorescence and red fluorescence indicate DPP activity and *E. coli* cells, respectively. The scale bar represents 100 μm.

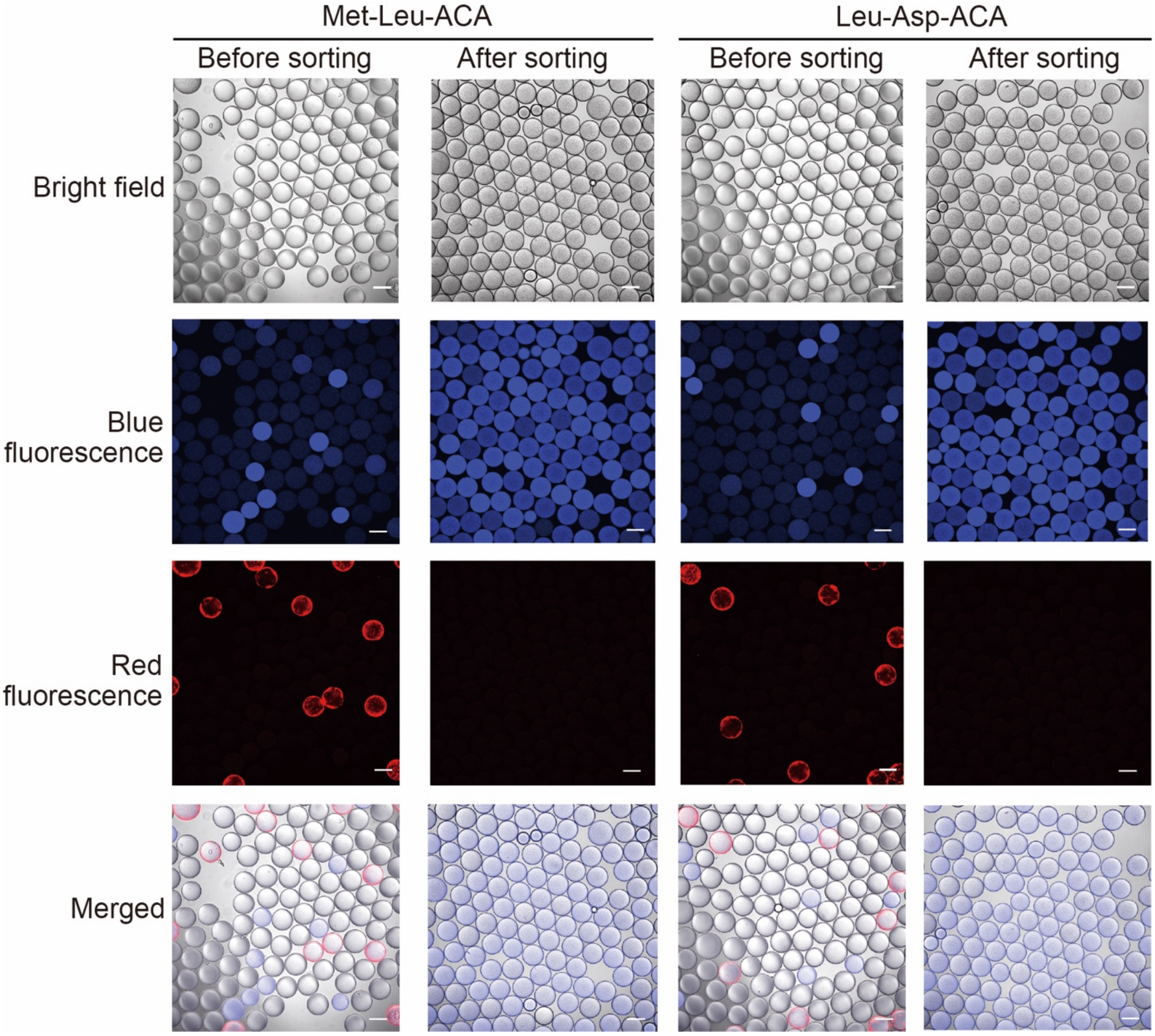

Figure S13 | Micrographs of high-throughput sorting of model microorganisms using dipeptidyl ACA substrates.

Droplet images were obtained by the confocal microscope A1 HD25 under 100× magnification. Blue fluorescence and red fluorescence indicate DPP activity and *E. coli* cells, respectively. The scale bar represents 100 μm.

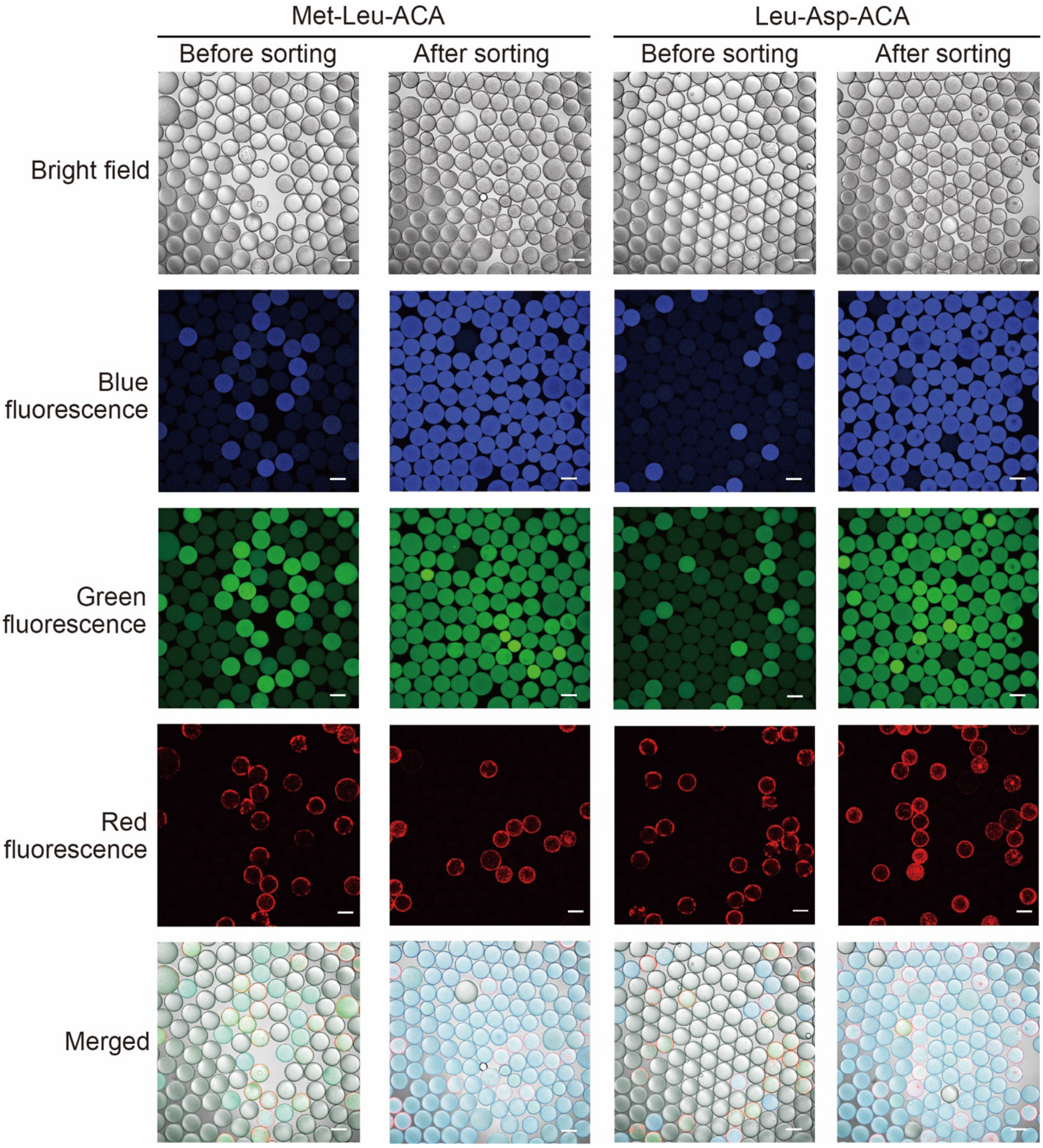

Figure S14 | Micrographs of high-throughput isolation based on DPP activity from suspensions containing two kinds of bacteria.

Droplet images were obtained by the confocal microscope A1 HD25 under 100× magnification. Blue fluorescence, green fluorescence, and red fluorescence indicate DPP activity, growth of microorganisms (RNase activity), and *E. coli* cells, respectively. The scale bar represents 100 μm.

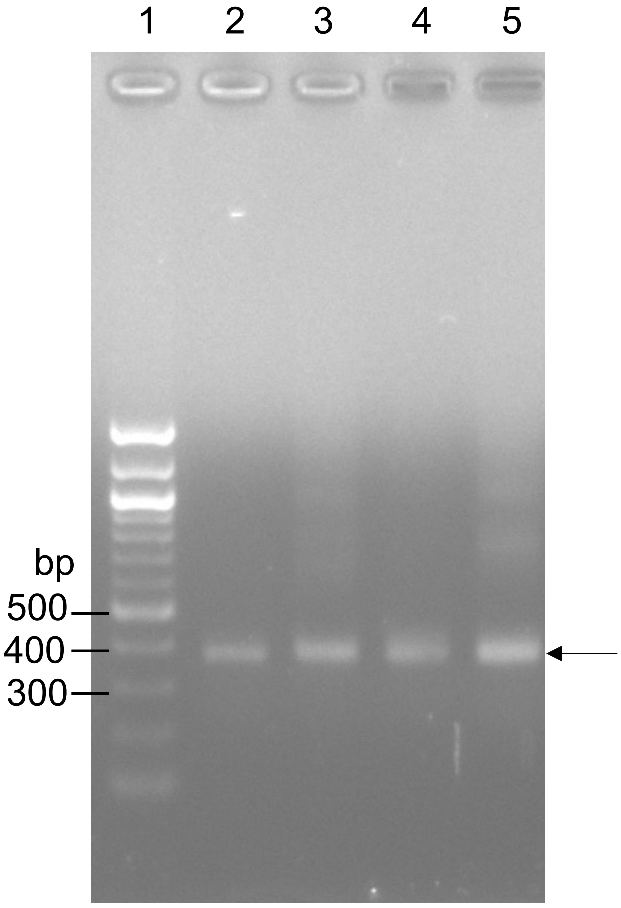

Figure S15 | Agarose-gel electrophoresis of an ante-sequencing 16S rDNA sample.

A 1.5% agarose gel was used, and 2 μL of amplified and purified 16S rDNA fragments were applied. Lane 1: 100 bp DNA Ladder (New England Biolabs, Japan). Lane 2: *tofu* factory waste fluid sample. Lane 3: sample after WODL cultivation. Lane 4: FADS (M-L-ACA activity) sample. Lane 5: FADS (L-D-ACA activity) sample.

Table S1 | The number of reads after quality filtering and OTUs.

Sample	Reads (after quality filtering)	OTUs
Tofu factory waste fluid	27 081	534
After WODL cultivation	32 906	349
FADS (M-L-ACA activity)	28 855	252
FADS (L-D-ACA activity)	31 344	318

Table S2 | Isolated microorganisms using dipeptidyl ACA substrates.

Sample specificity	No.	Determined strains using BLAST			Homology search using BLAST				
						Predicted substrate degradability			
		Strains	Identify (%)	Accession No.	Taxid	M-L-ACA L-D-ACA	M-L-ACA	M-L-ACA L-D-ACA	M-L-ACA L-D-ACA
						DPP3	DPP5	DPP7	DPP11
Common	1	*Sphingomonas yabuuchiae* strain A1-18	99.7	NR_028634.1	172044	-	-	-	-
	2	*Pseudoduganella danionis* strain E3/2	98.2	NR_152711.1	1890295	-	○	○	○
	3	*Stenotrophomonas rhizophila* strain e-p10	99.0	NR_121739.1	216778	-	○	○	○
	4	*Chryseobacterium camelliae* strain THG C4-1	95.2	NR_133724.1	1265445	-	○	○	○
	5	*Flavobacterium chilense* strain LM-09-Fp	99.6	NR_108512.1	946677	-	○	○	○
	6	*Aeromonas hydrophila* strain ATCC 7966	99.7	NR_074841.1	644	-	○	-	-
M-L-ACA specific	7	*Enterobacter soli* ATCC BAA-2102 strain LF7	99.4	NR_117547.1	885040	-	-	-	-
	8	*Falsarthrobacter* (*Arthrobacter*) *nasiphocae* strain M597/99/10	98.1	NR_025424.1	1663	-*	○*	-*	-*
	9	*Janthinobacterium agaricidamnosum* strain NBRC 102515	99.4	NR_114134.1	55508	-	○	○	○
	10	*Stenotrophomonas terrae* strain R-32768	99.0	NR_042569.1	405446	-	○	○	○
	11	*Lactococcus lactis* strain NBRC 100933	99.8	NR_113960.1	1358	-	-	-	-
	12	*Pantoea agglomerans* strain DSM 3493	99.2	NR_041978.1	549	-	-	-	-
	13	*Klebsiella grimontii* strain SB73	99.0	NR_159317.1	2058152	-	-	-	-
	14	*Cronobacter dublinensis* subsp. lausannensis strain E515	99.6	NR_044058.1	413497	-	-	-	-
	15	*Moraxella osloensis* strain DSM 6998	99.1	NR_113392.1	34062	-	-	-	-
	16	*Pseudomonas otitidis* strain MCC10330	99.8	NR_043289.1	319939	-	-	-	-
	17	*Brevundimonas faecalis* strain CS20.3	99.1	NR_117187.1	947378	-	-	-	-
	18	*Massilia violacea* strain CAVIO	99.8	NR_148592.1	1715466	-	○	○	○
L-D-ACA specific	19	*Aeromonas allosaccharophila* strain CECT 4199	99.7	NR_025945.2	656	-	-	-	-
	20	*Pseudomonas protegens* strain CHA0	99.8	NR_114749.1	380021	-	-	-	-
	21	*Chryseobacterium gallinarum* strain 100	99.5	NR_133726.1	1324352	-	○	○	○
	22	*Paenarthrobacter nitroguajacolicus* strain G2-1	99.8	NR_027199.1	211146	-	-	-	-
	23	*Chryseobacterium taihuense* strain THMBM1	97.6	NR_109542.1	1141221	-	○	○	○
	24	*Flavobacterium ginsengiterrae* strain DCY55	96.4	NR_132661.1	237	○*	○*	○*	○*
	25	*Pararheinheimera arenilitoris* strain J-MS1	98.1	NR_134151.1	152089	-	-	-	-
	26	*Chryseobacterium arachidis* strain 91A-593	98.5	NR_133723.1	1416778	-	○	○	○
	27	*Sphingomonas trueperi* strain NBRC 100456	99.9	NR_113897.1	53317	-	○	○	-
	28	*Pseudomonas japonica* NBRC 103040	98.9	NR_114192.1	256466	-	○	-	-
	29	*Cloacibacterium haliotis* strain WB5	98.6	NR_125655.1	501783	-*	○*	○*	○*
	30	*Brevundimonas terrae* strain KSL-145	99.4	NR_043726.1	363631	-	○	○	○

The strains were identified from the sequences containing the variable regions V1–V5 of the 16S rRNA gene, using the Basic Local Alignment Search Tool (BLAST).[5] The 16S ribosomal RNA sequences (Bacteria and Archaea) database was used, and top hit strains are listed in this

table. A homology search was performed using BLAST[5,6] to determine whether each species (not strains) carries the DPP gene, based on the following amino acid sequences. DPP3 from *Porphyromonas gingivalis* (UniProtKB: B2RLB9), DPP5 from *P. gingivalis* (UniProtKB: B2RIT0), DAP BII (UniProtKB: V5YM14), and PgDPP11 (UniProtKB: B2RID1) were used as the query sequence for DPP3, DPP5, DPP7, and DPP11, respectively. The specificity of the P1 position is as follows. DPP3: arginine, aliphatic, and acidic (degradable but low activity) amino acids.[7] DPP5: aromatic and aliphatic amino acid.[7] Family S46 peptidases DPP7: aromatic, aliphatic, and acidic (degradable but low activity) amino acid.[8] Family S46 peptidases DPP11: aliphatic and acidic amino acid.[9,10] A circle means possessing DPP homolog genes with over 30% of sequence homology. *Since there were no taxonomy IDs for species in BLAST, we performed homology searches using taxonomy IDs for genera.

Supplemental Materials and Methods

Materials and General Procedures for substrate synthesis.

Reagents and solvents used were purchased from BLD Pharm (CN), FUJIFILM Wako Pure Chemical Co., Ltd. (Japan), Nacalai Tesque (Japan), and Watanabe Chemical Industries, Ltd. (Japan), and used without further purification. All synthetic compounds were purified by preparative HPLC with > 95% purity and identified by Time-of-Flight Mass Spectrometry (TOF-MS). Analytical HPLC was performed using a C18 reversed-phase column (4.6 × 250 mm; Cosmosil $5C_{18}$-AR-II) with binary solvent systems: a linear gradient of CH_3CN 10–90% in 0.05% aqueous TFA for 40 min at a flow rate of 1.0 mL/min, detected at 220 nm. Preparative HPLC was carried out on a C18 reversed-phase column (20 × 250 mm; Cosmosil $5C_{18}$-AR-II) with a binary solvent system: a linear gradient of CH_3CN in 5 mM aqueous hydrochloric acid with a flow rate of 5.0 mL/min and detection at 220 nm. Proton nuclear magnetic resonance (^{1}H NMR) spectra were recorded on DPX400 (Bruker, Co., MA, USA) spectrometers with tetramethylsilane (TMS) as an internal standard. Mass spectra with electrospray ionization, with 50% aqueous methanol as the mobile phase, were obtained from a micrOTOF-Q II spectrometer (Bruker, Co., MA, USA).

Synthesis of coumarins and substrates.

2-(7-((((9*H*-Fluoren-9-yl)methoxy)carbonyl)amino)-2-oxo-2H-chromen-4-yl)acetic acid (Fmoc-ACA, 1): To a solution of 2-(7-amino-2-oxo-2*H*-chromen-4-yl)-acetic acid (241 mg, 1.1 mmol) and sodium bicarbonate (92.4 mg, 1.1 mmol) in water (5.5 mL) with acetone was added 9-fluorenylmethl chloroformate (259 mg, 1.0 mmol). The mixture was stirred overnight at room temperature. After removal of the solvent in vacuo, the residue was added into 1 M hydrochloric acid. The precipitate was filtered and washed with water and hexane to give the crude product. Precipitation with ether, filtration, and additional washing with 1 M hydrochloric acid and hexane yielded compound 1 with the following characteristics: a yellowish-white solid; yield 71%; ^{1}H NMR (400 MHz, DMSO-d_6) δ ppm 12.80 (br. s., 1 H), 10.21 (s, 1 H), 7.92 (d, J = 7.46 Hz, 2 H), 7.76 (d, J = 7.46 Hz, 2 H), 7.62 (d, J = 8.68 Hz, 1 H), 7.55 (br. s., 1 H), 7.46–7.34 (m, 5 H), 6.39–6.32 (m, 1 H), 4.57 (d, J = 6.36 Hz, 2 H), 4.34 (t, J = 6.42 Hz, 1 H), 3.86 (s, 2 H); MS (electrospray ionization (ESI)-TOF) *m*/*z*: calculated for $C_{26}H_{20}NO_6$ $[M + H]^+$ 442.1285; found 442.1268.

Dipeptidyl ACA substrates: Peptides containing ACA were prepared by conventional solid phase peptide synthesis using Fmoc chemistry. A mixture of 2-chlorotrityl chloride resin (354 mg, 0.54 mmol), 1 (309 mg, 0.70 mmol), *N,N*-diisopropylethylamine (177 µL, 1.0 mmol) in dry dichloromethane (2 mL) was vortexed overnight (loading yield, 60%). The resin (173 mg, 0.11 mmol) was treated with 20% piperidine in *N,N′*-dimethylformamide (DMF) for 20 min at room temperature. Fmoc-amino acid (0.54 mmol), 1-[bis(dimethylamino)methylene]-1H-1,2,3-triazolo[4,5-b]pyridinium 3-oxide hexafluorophosphate (HATU) (207 mg, 0.54 mmol), and 2,4,6-collidine (72 µL, 0.54 mmol) in DMF (1.5 mL) were added to the resin, and vortexed overnight at room temperature. The Fmoc group was deprotected with 20% piperidine in DMF for 20 min. A mixture of 1-hydroxybenzotriazole (HOBt) (50 mg, 0.33 mmol), Fmoc-amino acid (0.33 mmol), and *N,N′*-diisopropylcarbodiimide (DIC) (51 µL, 0.33 mmol) in DMF (1 mL) was added to the resin and vortexed for 2 h, and the subsequent deprotection was performed with 20% piperidine in DMF. The resins were stirred with trifluoroacetic acid (TFA)/triisopropylsilane (TIS)/H_2O/3,6-dioxa-1,8-octane-dithiol (DODT) (92.5:2.5:2.5:2.5) for peptide 2 or TFA/TIS/H_2O (95:2.5:2.5) for peptide 3 for 2 h at room temperature. Ether precipitation resulted in a crude white solid. Purification using preparative RP-HPLC gave the desired peptides as white powders.

2-(7-((S)-2-((S)-2-Amino-4-(methylthio)butanamido)-4-methylpentanamido)-2-oxo-2H-chromen-4-yl)acetic acid hydrochloride (Met-Leu-ACA, 2): Yield 10.6 mg (19%); purity 98.3%; ^{1}H NMR (400 MHz, DMSO-d_6) δ ppm 10.71 (s, 1 H), 8.80 (d, *J* = 7.46 Hz, 1 H), 8.13 (br. s., 3H), 7.81–7.77 (m, 1 H), 7.66–7.76 (m, 1 H), 7.53–7.47 (m, 1 H), 6.38 (s, 1 H), 4.52 (ddd, *J* = 9.63, 7.31, 5.20 Hz, 1 H), 4.00–3.72 (m, 3 H), 2.57–2.53 (m, 1 H), 2.53 (br. s., 1 H), 2.46 (dd, *J* = 3.67, 1.83 Hz, 1 H), 2.40 (d, *J* = 1.22 Hz, 1 H), 2.12–1.92 (m, 5 H), 1.75–1.52 (m, 3 H), 0.94 (t, *J* = 6.85 Hz, 6 H); MS (ESI-TOF) *m*/*z*: calculated for $C_{22}H_{30}N_3O_6S$ $[M + H]^+$ 464.1850; found 464.1833.

(S)-3-((S)-2-Amino-4-methylpentanamido)-4-((4-(carboxymethyl)-2-oxo-2H-chromen-7-yl)amino)-4-oxobutanoic acid hydrochloride (Leu-Asp-ACA, 3): Yield 6.3 mg (12%), purity 95.8%; ^{1}H NMR (400 MHz, DMSO-d_6) δ ppm 10.70 (s, 1 H), 8.96 (d, *J* = 7.09 Hz, 1 H), 8.20 (br. s., 3H), 7.82–7.75 (m, 1 H), 7.75–7.64 (m, 1 H), 7.54–7.44 (m, 1 H), 6.38 (s, 1 H), 6.29 (d, *J* = 1.34 Hz, 1 H), 4.80–4.71 (m, 1 H), 3.88 (s, 2 H), 3.77 (dd, *J* = 8.31, 5.99 Hz, 1 H), 2.88–2.81

(m, 1 H), 2.70–2.62 (m, 2 H), 2.52 (br. s., 1 H), 2.40 (d, *J* = 1.22 Hz, 1 H), 1.76–1.49 (m, 4 H), 0.94–0.84 (m, 6 H); MS (ESI-TOF) *m/z*: calculated for $C_{21}H_{26}N_3O_8$ $[M + H]^+$ 448.1714; found 448.1705.

(*S*)-2-((*S*)-2-((*tert*-Butoxycarbonyl)amino)-4-(methylthio)butanamido)-4-methylpentanoic acid (Boc-Met-Leu-OH, 4): To a solution of leucine methyl ester hydrochloride (73.9 mg, 0.4 mmol) in DMF (5 mL), *N,N,N*-triehthylamine (55.9 µL, 0.4 mmol), HOBt monohydrate (67.4 mg, 0.44 mmol), Boc-Met-OH (110 mg, 0.44 mmol), and *N*-Ethyl-*N'*-(3-dimethylaminopropyl)carbodiimide hydrochloride (EDC) were added. The mixture was stirred overnight at room temperature. After removal of the solvent in vacuo, the residue was added into ethyl acetate, washed sequentially with 10% citric acid, 5% sodium bicarbonate, and saturated sodium chloride, dried with sodium sulfate, and filtered. Removal of the ethyl acetate in vacuo gave a white solid as an intermediate (137 mg). To a solution of the filtered solid in methanol (2 mL), 1 M NaOH aq. (2 mL) was added. It was stirred for two hours at room temperature. After removal of the methanol in vacuo, the solution was acidified by citric acid, then extracted with ethyl acetate, washed with brine, dried with sodium sulfate, and filtered. After removal of the solvent in vacuo, precipitation with ether gave the titled compound as a white solid (143 mg); yield 143 mg 99%; ^{1}H NMR (400 MHz, DMSO-d_6) δ ppm 12.44 (br. s., 1 H), 8.00 (d, *J* = 7.95 Hz, 1 H), 6.94 (d, *J* = 8.19 Hz, 1 H), 4.22 (ddd, *J* = 9.75, 7.98, 5.26 Hz, 1 H), 4.09–3.93 (m, 1 H), 2.77–2.67 (m, 1 H), 2.67–2.57 (m, 1 H), 2.44 (t, *J* = 7.89 Hz, 2 H), 2.03 (s, 3 H), 1.88–1.71 (m, 2 H), 1.71–1.60 (m, 1 H), 1.60–1.43 (m, 2 H), 1.37 (s, 9 H), 0.89 (d, *J* = 6.60 Hz, 3 H), 0.83 (d, *J* = 6.48 Hz, 3 H); MS (ESI-TOF) *m/z*: calculated for $C_{16}H_{31}N_2O_5S$ $[M + H]^+$ 363.1948; found 363.1938.

(7-((*S*)-2-((*S*)-2-Amino-4-(methylthio)butanamido)-4-methylpentanamido)-2-oxo-2H-chromen-4-yl)methanesulfonic acid hydrochloride (Met-Leu-ACMS, 5): To a solution of intermediate 4 (121 mg, 0.30 mmol) in dry DMF (4 mL), *N*-methylmorpholine (33.0 µL, 0.30 mmol), and isobutyl chloroformate (38.9 µL, 0.30 mmol) were added at –10 ºC in an ice-salt bath. The mixture was stirred for 30 min. A solution of (7-amino-2-oxo-2H-chromen-4-yl)methanesulfonic acid[2] (69.7 mg, 0.27 mmol) and *N*-methylmorpholine (150 µL, 1.37 mmol) in dry DMF (1 mL) was added and stirred overnight at room temperature. After removal of the solvent in vacuo, the residue was added into ethyl acetate, washed sequentially with 1M HCl aq. and brine, dried with sodium sulfate, filtered, and

evaporated to give an intermediate as a slightly yellow-colored oil. The solid was dissolved in 4M HCl/ethyl acetate (2 mL) with anisole (65.2 µL, 0.6 mmol) and stirred for one hour at room temperature. After removal of the solvent in vacuo, the residue was added to ether. The precipitate was filtered, dissolved in water, and lyophilized. Purification using preparative RP-HPLC gave the desired peptides as white powders: yield 0.64 mg; purity 96.3%; ^{1}H NMR (400 MHz, DMSO-d_6) δ ppm 10.65 (s, 1 H), 8.89 (br. s., 1 H), 7.91 (d, J = 8.80 Hz, 1 H), 7.82 (d, J = 2.08 Hz, 1 H), 7.42–7.40 (m, 1 H), 6.27 (s, 1 H), 4.63–4.54 (m, 1 H), 4.08–3.93 (m, 2 H), 3.90–3.82 (m, 1 H), 2.62–2.59 (m, 2 H), 2.09–1.87 (m, 5 H), 1.73–1.48 (m, 3 H), 0.94 (d, J = 6.11 Hz, 3 H), 0.91 (d, J = 6.24 Hz, 3 H); MS (ESI-TOF) *m/z*: calculated for $C_{21}H_{30}N_3O_7S_2$ $[M + H]^+$ 500.1520; found 500.1489.

Preparation of 16s rDNA library and sequencing.

For metagenomic analysis, genomic DNA from each sample except the WODL sample was extracted using the FastDNA Spin Kit for Soil (MP Biomedicals, Santa Ana, CA). Genomic DNA from the WODL sample was extracted by an original method as follows: The WODLs were broken using Pico-Break (Sphere Fluidics Limited, UK) and centrifuged at 6,000 × g. The pellet was washed by 0.9% NaCl solution and suspended in 700 µl of 6 mg/ml lysozyme in a lysis buffer consisting of 100 mM Tris-HCl (pH 8.0), 100 mM EDTA, and 1.5 M NaCl. The suspension was incubated overnight at 37 °C. Next, 10 µL of 10 mg/mL proteinase K (Nacalai Tesque, Inc., Kyoto, Japan), 10 µl of 10 mg/ml RNase (Sigma Aldrich, USA), and 70 µl of 10% (w/v) sodium dodecyl sulfate (Nacalai Tesque, Inc., Kyoto, Japan) were added to the suspension and shaken at 55 °C for 30 min. After centrifuging at 20,000 × g, 700 µl of supernatant was mixed with phenol/chloroform (Nacalai Tesque, Inc., Kyoto, Japan). After centrifugation at 20,000 × g, 500 µL of supernatant was collected in a new Eppendorf tube, and 50 µL of 3 M CH_3COONa and 1,250 µL of 100% ethanol were added to the supernatant. The supernatant was removed after centrifugation at 20,000 × g, and the pellet was washed by 1 mL of 70% (v/v) ethanol. After centrifugation at 20,000 × g, the pellet was dried and dissolved in a TE buffer consisting of 10 mM Tris-HCl (pH 8.0) and 1 mM EDTA. A forward universal bacterial primer 515F (5′-GTGCCAGCMGCCGCGGTAA-3′) and a reverse universal primer 806R (5′-GGACTACHVGGGTWTCTAAT-3′) were used to amplify the bacterial 16S rRNA genes. Polymerase chain reaction (PCR) was performed using KOD FX Neo (Toyobo Co., Ltd., Osaka, Japan) as a DNA polymerase. Conditions were set as follows: one cycle of 94 °C for 2

min, 40 cycles of 98 °C for 10 sec, and 68 °C for 30 sec. PCR products were purified using a MinElute PCR purification kit (Qiagen, Germany) (Figure S15), and the PCR product concentrations were measured with a Qubit2.0 Fluorometer (Thermo Fisher Scientific, MA, USA). The 16S rRNA gene sequencing was performed as described in the literature.[11] DNA was sequenced using a MiSeq Reagent Kit v2 and the MiSeq System (Illumina Inc., CA, USA). For isolated microorganisms, between 27 bp and 1,500 bp of 16S rRNA genes were amplified using forward primer 27F (5′-AGAGTTTGATCATGGCTCAG-3′) and reverse primer 1,500R (5′-TACCTTGTTACGACTT-3′), and each colony was used as a template. PCR was performed using KOD FX Neo (Toyobo Co., Ltd., Osaka, Japan) as a DNA polymerase. Conditions were set as follows: one cycle of 94 °C for 2 min, 35 cycles of 98 °C for 10 sec, 55 °C for 30 sec, and 68 °C for 2 min. PCR products were sequenced by the Sanger method carried out by Eurofins Genomics (Tokyo, Japan).

References in supporting information

(1) Schneider, C. A.; Rasband, W. S.; Eliceiri, K. W. NIH Image to ImageJ: 25 Years of Image Analysis. *Nat. Methods* **2012**, *9* (7), 671–675.

(2) Mazutis, L.; Gilbert, J.; Ung, W. L.; Weitz, D. A.; Griffiths, A. D.; Heyman, J. A. Single-Cell Analysis and Sorting Using Droplet-Based Microfluidics. *Nat. Protoc.* **2013**, *8* (5), 870–891.

(3) Goddard, T. D.; Huang, C. C.; Meng, E. C.; Pettersen, E. F.; Couch, G. S.; Morris, J. H.; Ferrin, T. E. UCSF ChimeraX: Meeting Modern Challenges in Visualization and Analysis. *Protein Sci.* **2018**, *27* (1), 14–25.

(4) LAGUERRE, M.; SAUX, M.; DUBOST, J. P.; CARPY, A. MLPP: A Program for the Calculation of Molecular Lipophilicity Potential in Proteins. *Pharm. Pharmacol. Commun.* **1997**, *3* (5-6), 217–222.

(5) Altschul, S. F.; Madden, T. L.; Schäffer, A. A.; Zhang, J.; Zhang, Z.; Miller, W.; Lipman, D. J. Gapped BLAST and PSI-BLAST: A New Generation of Protein Database Search Programs. *Nucleic Acids Res.* **1997**, *25* (17), 3389–3402.

(6) Altschul, S. F.; Wootton, J. C.; Gertz, E. M.; Agarwala, R.; Morgulis, A.; Schäffer, A. A.; Yu, Y.-K. Protein Database Searches Using Compositionally Adjusted Substitution Matrices. *FEBS J.* **2005**, *272* (20), 5101–5109.

(7) Ohara-Nemoto, Y.; Rouf, S. M. A. A.; Naito, M.; Yanase, A.; Tetsuo, F.; Ono, T.; Kobayakawa, T.; Shimoyama, Y.; Kimura, S.; Nakayama, K.; Saiki, K.; Konishi, K.; Nemoto, T. K. Identification and Characterization of Prokaryotic Dipeptidyl-Peptidase 5 from *Porphyromonas gingivalis*. *J. Biol. Chem.* **2014**, *289* (9), 5436–5448.

(8) Nishimata, H.; Ohara-Nemoto, Y.; Baba, T. T.; Hoshino, T.; Fujiwara, T.; Shimoyama, Y.; Kimura, S.; Nemoto, T. K. Identification of Dipeptidyl-Peptidase (DPP)5 and DPP7 in *Porphyromonas endodontalis*, Distinct from Those in *Porphyromonas gingivalis*. *PLoS One* **2014**, *9* (12), e114221.

(9) Sakamoto, Y.; Suzuki, Y.; Iizuka, I.; Tateoka, C.; Roppongi, S.; Fujimoto, M.; Inaka, K.; Tanaka, H.; Yamada, M.; Ohta, K.; Gouda, H.; Nonaka, T.; Ogasawara, W.; Tanaka, N. Structural and Mutational Analyses of Dipeptidyl Peptidase 11 from *Porphyromonas*

gingivalis Reveal the Molecular Basis for Strict Substrate Specificity. *Sci. Rep.* **2015**, *5* (1), 11151.

(10) Nakamura, A.; Suzuki, Y.; Sakamoto, Y.; Roppongi, S.; Kushibiki, C.; Yonezawa, N.; Takahashi, M.; Shida, Y.; Gouda, H.; Nonaka, T.; Tanaka, N.; Ogasawara, W. Structural Basis for an Exceptionally Strong Preference for Asparagine Residue at the S2 Subsite of *Stenotrophomonas maltophilia* Dipeptidyl Peptidase 7. *Sci. Rep.* **2021**, *11* (1), 7929.

(11) Caporaso, J. G.; Lauber, C. L.; Walters, W. A.; Berg-Lyons, D.; Huntley, J.; Fierer, N.; Owens, S. M.; Betley, J.; Fraser, L.; Bauer, M.; Gormley, N.; Gilbert, J. A.; Smith, G.; Knight, R. Ultra-High-Throughput Microbial Community Analysis on the Illumina HiSeq and MiSeq Platforms. *ISME J.* **2012**, *6* (8), 1621–1624.
